# Chloroplast‐inspired Scaffold for Infected Bone Defect Therapy: Towards Stable Photothermal Properties and Self‐Defensive Functionality

**DOI:** 10.1002/advs.202204535

**Published:** 2022-09-15

**Authors:** Yao Zhao, Xu Peng, Dingqian Wang, Hongbo Zhang, Qiangwei Xin, Mingzhen Wu, Xiaoyang Xu, Fan Sun, Zeyuan Xing, Luning Wang, Peng Yu, Jing Xie, Jiehua Li, Hong Tan, Chunmei Ding, Jianshu Li

**Affiliations:** ^1^ College of Polymer Science and Engineering State Key Laboratory of Polymer Materials Engineering Sichuan University Chengdu 610065 China; ^2^ Experimental and Research Animal Institute Sichuan University Chengdu 610065 China; ^3^ State Key Laboratory of Oral Diseases West China Hospital of Stomatology Med‐X Center for Materials Sichuan University Chengdu 610041 China

**Keywords:** antimicrobial ability, black phosphorus, chloroplasts, osteogenesis, self‐defensive

## Abstract

Bone implant‐associated infections induced by bacteria frequently result in repair failure and threaten the health of patients. Although black phosphorus (BP) material with superior photothermal conversion ability is booming in the treatment of bone disease, the development of BP‐based bone scaffolds with excellent photothermal stability and antibacterial properties simultaneously remains a challenge. In nature, chloroplasts cannot only convert light into chemical energy, but also hold a protective and defensive envelope membrane. Inspired by this, a self‐defensive bone scaffold with stable photothermal property is developed for infected bone defect therapy. Similar to thylakoid and stroma lamella in chloroplasts, BP is integrated with chitosan and polycaprolactone fiber networks. The mussel‐inspired polydopamine multifunctional “envelope membrane” wrapped above not only strengthens the photothermal stability of BP‐based scaffolds, but also realizes the in situ anchoring of silver nanoparticles. Bacteria‐triggered infection of femur defects in vivo can be commendably inhibited at the early stage via these chloroplast‐inspired implants, which then effectively promotes endogenous repair of the defect area under mild hyperthermia induced by near‐infrared irradiation. This chloroplast‐inspired strategy shows outstanding performance for infected bone defect therapy and provides a reference for the functionality of other biomedical materials.

## Graphical abstract

For infected bone defect therapy, developing black phosphorus (BP)‐based scaffolds with acceptable photothermal stability and antibacterial properties remains a challenge. Inspired by chloroplasts, a polydopamine multifunctional “envelope membrane” is induced to stabilize BP into the scaffold and synchronously grow Ag nanoparticles. The self‐defensive platform with pH‐responsive release of Ag^+^ and enhanced photothermal stability can achieve bacterial inhibition and photothermal osteogenesis in vivo.

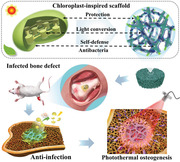

## 1 Introduction

Implant‐associated infections remain a serious problem in bone defect therapy and frequently result in repair failure, potentially threatening the health of patients.^[^ ^1^, ^2^ ^]^ Recently, implanted scaffolds loaded with various antibiotics,^[^ ^3^, ^4^ ^]^ such as ciprofloxacin and gentamicin, or antimicrobial nanoparticles,^[^ ^5^ ^]^ such as gold and silver, have gradually become a research hotspot. Nevertheless, antibiotic resistance, disquieting cytotoxicity of over‐released nanoparticles, and weak osteo‐integration and osteo‐inductivity of the scaffold are still limitations of these implantations.^[^ ^2^, ^6^ ^]^ Hence, developing bioactive scaffolds with satisfactory osteogenic potential and antimicrobial ability is still a challenge for the treatment of bone implant‐associated infections.

Photothermal therapy has proven to be an efficient strategy to improve the osteogenesis of implants.^[^ ^7^, ^8^, ^9^ ^]^ Over the last few years, advances have been made by integrating carbon nanotubes^[^ ^10^ ^]^ or graphene oxide^[^ ^11^ ^]^ with scaffolds to facilitate osteogenesis. Nevertheless, the biotoxicity and immune responses of these inorganic particles remain controversial.^[^ ^12^ ^]^ Recently, black phosphorus (BP) nanosheets, which are promising 2D nanomaterials, have been widely researched and applied in the field of photothermal therapy^[^ ^13^, ^14^ ^]^ owing to their high photothermal conversion efficiency and biological activities.^[^ ^8^, ^15^ ^]^ Besides, the degradation products of BP provide phosphorus sources for bone regeneration.^[^ ^16^, ^17^, ^18^ ^]^ Unfortunately, the interaction between BP and the scaffold matrix is weak, and BP is degraded easily under physiological conditions, which weakens the thermal properties and limits its application in long‐term photothermal therapy of bone repair.^[^ ^14^, ^19^ ^]^ For this reason, it is necessary to stabilize BP in a scaffold to achieve persistent photothermal stability. However, stabilizing BP in bone repair scaffolds and simultaneously endowing the scaffold with good antimicrobial properties has not yet been achieved.

The chloroplast provides hints for the design of therapeutic scaffolds with photothermal stability and antimicrobial ability. As a sunlight‐driven metabolic organelle in higher plants, chloroplasts maintain the normal life activities of all organisms on earth.^[^ ^20^ ^]^ Therein, thylakoids, which are interconnected through a stroma lamella network, are responsible for light‐harvesting and energy‐transducing via photosynthetic pigment molecules on their surface.^[^ ^21^ ^]^ Strikingly, the envelope membrane (lipid layer) of chloroplasts has special physical and biological functions. The envelope membrane acts as a natural barrier that can protect the structural integrity inside the chloroplast.^[^ ^22^ ^]^ Furthermore, special proteins, such as EDS5, localized on the envelope membrane, are responsible for the biosynthesis and transportation of immunity‐related salicylic acid from chloroplasts to the cytoplasm.^[^ ^23^ ^]^ Therefore, the envelope membrane of plant chloroplasts can adaptively defend against pathogens, such as bacterial infections and viruses.

**Scheme 1:**
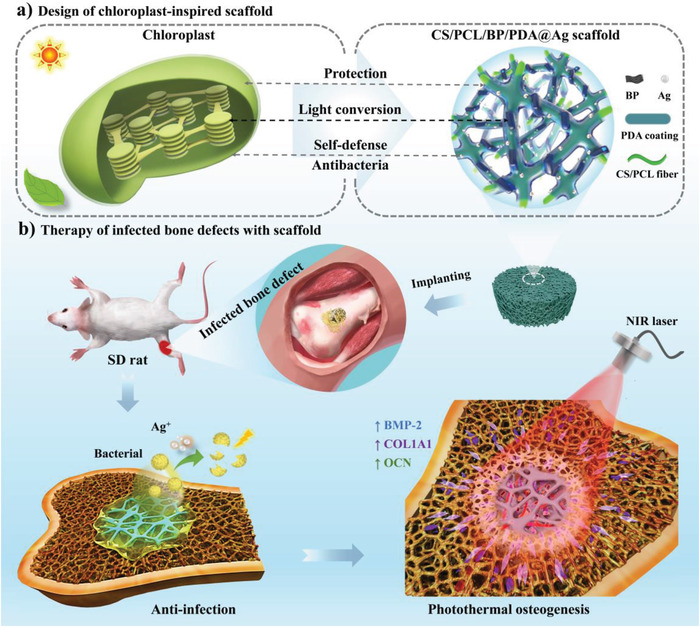
Schematic illustration of the chloroplast‐inspired CS/PCL/BP/PDA@Ag scaffold for therapy of infected bone defect. a) The chloroplast‐inspired functional design for the CS/PCL/BP/PDA@Ag scaffold (similar protection, light conversion and self‐defense functions between the chloroplast and CS/PCL/BP/PDA@Ag scaffold). b) Chloroplast‐inspired CS/PCL/BP/PDA@Ag scaffold with excellent self‐defensive functionality and photothermal osteogenesis for infected bone defect therapy.

Hence, inspired by the functions of protection, light conversion and self‐defense of natural chloroplasts, BP nanosheets serve as thylakoids to capture near‐infrared (NIR) light and convert it to heat, while 3D porous chitosan and polycaprolactone (CS/PCL) composite fiber networks similar to the interlinked stroma lamella are used to interconnect these BP nanosheets. To further endow the BP‐based scaffold with practical stability and self‐defensive functionality, a mussel‐inspired polydopamine (PDA) multifunctional “envelope membrane” with rich catechol groups^[^ ^24^ ^]^ was induced to stabilize BP nanosheets into the scaffold and synchronously realize in situ growth and anchoring of the antimicrobial agent of silver (Ag) nanoparticles.^[^ ^25^ ^]^ Ultimately, a chloroplast‐inspired 3D CS/PCL/BP/PDA@Ag scaffold was successfully developed (**Scheme** **1**a). The bioinspired platform with expectant self‐defensive functionality and photothermal stability was used to achieve bacterial inhibition and photothermal osteogenesis in vivo (Scheme 1b).

## 2 Results and Discussion

### 2.1 Fabrication and Characterization of the Chloroplast‐Inspired Scaffolds

**Figure 1:**
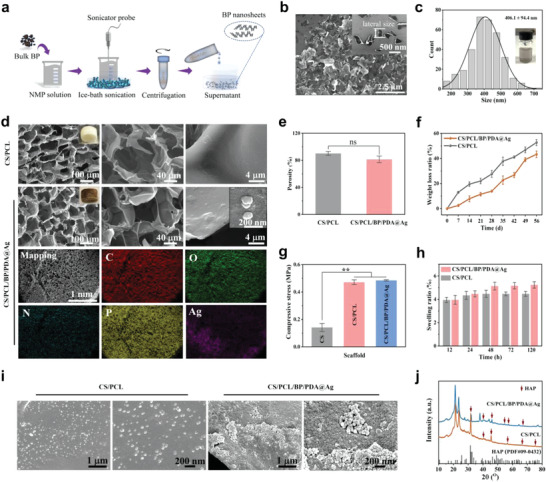
Characterizations of chloroplast‐inspired CS/PCL/BP/PDA@Ag scaffold. a) Schematic diagram illustrating the preparation of BP nanosheets. b) The scanning electron microscopy (SEM) images of BP nanosheets and a high magnification image in the inset (scale bar = 500 nm). c) Statistical analysis of the lateral size of BP sheets based on the SEM images (inset: uniform aqueous dispersion of BP). d) The fractured cross‐section SEM images of the CS/PCL and CS/PCL/BP/PDA@Ag scaffolds and corresponding element mappings of CS/PCL/BP/PDA@Ag scaffold. Insets show the macroscopic photographs of the corresponding scaffold and Ag nanoparticles (scale bar = 200 nm). e) Porosity of the CS/PCL and CS/PCL/BP/PDA@Ag scaffolds. f) The degradation curve of scaffolds in vitro after immersion in phosphate‐buffered saline solution (PBS) with enzyme at the presupposed time intervals. g) Compressive stress and h) swelling ratio of the CS/PCL and CS/PCL/BP/PDA@Ag scaffolds. i) SEM images and j) XRD patterns of the scaffolds after biomineralization in simulated body fluid (SBF) for 5 days. Error bars: mean ± SD (*n* = 5), ^**^ *p* < 0.01, ns denotes no significance.

BP nanosheets, serving as thylakoids in chloroplasts, were prepared by a liquid exfoliation method (**Figure** **1**a), and showed a typical 2D sheet structure (Figure 1b) with a lateral size of 406.1 ± 94.4 nm (Figure 1c). The as‐prepared BP nanosheets were well‐dispersed in water, indicating good hydrophilicity (inset in Figure 1c). Freeze‐dried CS/PCL composite fiber networks were then functionalized in sequence by the integration of BP nanosheets, modification with a PDA “envelope membrane,” and loading of Ag nanoparticles in situ. The color of the CS/PCL scaffold changed from white to brownish black after subsequent functionalization (Figure 1d). Both scaffolds had a 3D porous structure with pore sizes ranging from 20 to 200 µm, which is analogous to natural bone.^[^ ^26^ ^]^ In addition, Ag nanoparticles grown in situ had a diameter of ≈100 nm (as shown in the inset with a rectangular dashed line). It could be seen from both fractured cross section and surface images (Figure S1a, Supporting Information) of the porous scaffold that the functionalized scaffold generated a coarser surface of CS/PCL/BP/PDA@Ag than that of the CS/PCL scaffold, which benefited cellular adhesion and proliferation.^[^ ^27^ ^]^ The presence of C, O, N, P, and Ag elements was also suggestive of the successful integration of the scaffold with BP and Ag nanoparticles. Fourier transform infrared spectroscopy (FTIR), X‐ray diffraction (XRD), X‐ray photoelectron spectroscopy (XPS), and Raman spectra (Figures S1b–d and S2, Supporting Information) conjoint analysis further proved that the CS/PCL/BP/PDA@Ag scaffold was successfully fabricated (detailed analysis is described in the Supporting Information).

For bone tissue‐engineered scaffolds, it is necessary to investigate the properties that potentially effect new tissue growth, such as porosity, degradation, mechanical properties, hydrophilicity, swelling ratio, water uptake/retention ratio, and biomineralization in vitro. The porosity of both the CS/PCL and CS/PCL/BP/PDA@Ag scaffolds was over 81%, suggesting that they can provide enough space for the survival of cells (Figure 1e). There was no significant difference in porosity between the CS/PCL and CS/PCL/BP/PDA@Ag scaffolds, implying that the modification of BP/PDA@Ag had little effect on the structure of porous scaffold. The degradation behaviors of both scaffolds exhibited time‐dependent regularity (Figure 1f). In contrast, the weight loss ratio of CS/PCL/BP/PDA@Ag scaffolds was lower than that of the CS/PCL scaffolds during the entire test period. The maximal weight loss ratio can be detected on CS/PCL/BP/PDA@Ag scaffolds on the 49th day, whereas it is on the 35th day for CS/PCL scaffolds. The delayed degradation of the CS/PCL/BP/PDA@Ag scaffold might be due to the presence of the PDA coating, which effectively reduced the contact areas between the lipase and PCL fibers. The enhanced stability of CS/PCL/BP/PDA@Ag scaffolds prevents quick collapse under physiological conditions of bone implantation and is favorable for the bone‐implant integration in a long repair process.^[^ ^28^ ^]^

The mechanical properties of the CS scaffold were not satisfactory with a compressive stress of 0.141 ± 0.029 MPa and elastic modulus of 0.284 ± 0.073 MPa. After the incorporation of PCL, a 3.4‐ and 2.5‐fold enhancement for compressive stress and elastic modulus respectively can be achieved (Figure 1g; Figure S3, Supporting Information). Notably, the strong chelation and hydrogen bonding force among the PDA coating, nanoparticles, and PCL/CS fibers generated the highest compressive stress (0.48 ± 0.005 MPa) and elastic modulus (1.61 ± 0.15 MPa) of the CS/PCL/BP/PDA@Ag scaffold, providing a relatively stable biomechanical microenvironment for cellular growth in vivo.^[^ ^28^ ^]^

After the introduction of functional moieties (PDA coating, BP, and Ag nanoparticles), the hydrophilicity of the obtained CS/PCL/BP/PDA@Ag scaffold was highly improved, which is typically beneficial for cell spreading (Figure S4a, Supporting Information). Additionally, excessive swelling changes have adverse effects on the wound during implantation, such as compression and malposition.^[^ ^28^ ^]^ Nevertheless, the swelling ratio of the CS/PCL/BP/PDA@Ag scaffold in PBS was <5.2% (Figure 1h), and showed no significant difference from that of the CS/PCL scaffold. Hence, there should be no negative impact on tissue repair. Simultaneously, the good hydrophilicity endows the CS/PCL/BP/PDA@Ag scaffold with excellent water uptake and water retention ratio (Figure S4b,c, Supporting Information), which is beneficial for cell growth, metabolite accumulation, and prevention of tissue dehydration.^[^ ^28^ ^]^

The biomineralization ability of scaffolds as a key factor in bone tissue engineering was studied by soaking the obtained scaffolds in SBF at 37 °C for 1 day and 5 days. On the first day, a few granular nanoparticles were randomly deposited on the surface of both scaffolds (Figure S4d, Supporting Information). Whereas, after 5 days of immersion, more aggregated granular nanoparticles were formed on the CS/PCL/BP/PDA@Ag scaffold than on the CS/PCL scaffold (Figure 1i). Compared to XRD patterns of unmineralized scaffolds, (Figure S1c, Supporting Information), the new characteristic peaks at 2*θ* = 31.8°, 40.4°, 45.6°, 54.6°, 57.3°, 66.4°, and 75.8° emerged after mineralization, which can be assigned to the (211), (221), (203), (104), (313), (422), and (215) crystallographic planes of hydroxyapatite (*JCPDF#09‐0432*), respectively (Figure 1j). This outstanding biomineralization ability might be ascribed to the degradation of BP and chelation of PDA to calcium ions^[^ ^29^ ^]^ and is expected to weaken immunogenicity and facilitate integration with host bone tissue during implantation in vivo.^[^ ^30^ ^]^

### 2.2 Photothermal Properties of the Chloroplast‐Inspired Scaffolds

The photothermal properties of the CS/PCL/BP/PDA scaffold were evaluated by immersing all of the scaffolds in PBS. The photothermal performance of the wet CS/PCL/BP/PDA scaffold was dependent on the concentration and laser power density, owing to the excellent photothermal conversion efficiency of BP (Figure S5, Supporting Information). Furthermore, the CS/PCL/BP/PDA scaffolds showed good cell activity and proliferative potential when co‐cultured with MC3T3‐E1 cells (Figure S6, Supporting Information). According to the above results, the CS/PCL/BP/PDA scaffold with 100 ppm BP exhibited acceptable photothermal properties and good cytocompatibility, and was chosen for subsequent experiments.

**Figure 2:**
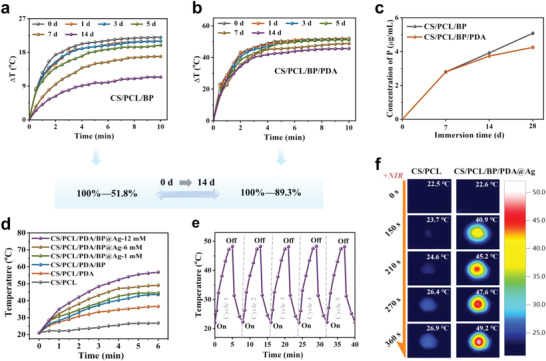
Photothermal properties of the chloroplast‐inspired scaffolds in vitro. Photothermal curves of a) the CS/PCL/BP scaffold and b) CS/PCL/BP/PDA scaffold after the immersion in water for different periods of time and irradiation with an 808 nm laser (1.0 W cm^−2^) for 10 min. c) Cumulative release curves of P element from CS/PCL/BP scaffold with or without PDA coating in water for 7, 14, and 28 days. d) Photothermal heating curves of the CS/PCL, CS/PCL/PDA, CS/PCL/BP/PDA, and CS/PCL/BP/PDA@Ag scaffolds with various initial AgNO_3_ concentrations irradiated with an NIR laser (808 nm, 0.5 W cm^−2^). e) Temperature variation of the CS/PCL/BP/PDA@Ag‐6 mm scaffold with repeated on‐off NIR irradiation (BP: 100 ppm, 808 nm, 0.5 W cm^−2^) for 5 cycles. f) Infrared thermographic maps of the CS/PCL and CS/PCL/BP/PDA@Ag scaffold under NIR laser irradiation for various irradiation time periods (BP:100 ppm).

In this work, a PDA “envelope membrane” was skillfully utilized to protect and passivate the BP in the scaffold so as to enhance the durable photothermal stability of the BP‐based scaffold. Consequently, the photostability of the CS/PCL/BP and CS/PCL/BP/PDA scaffolds was studied upon NIR laser irradiation (808 nm, 1 W cm^−2^) after soaking in air‐exposed water for different time intervals (**Figure** **2**a,b). Initially, the solution temperature of the CS/PCL/BP scaffold increased by 22 °C after NIR laser irradiation for 10 min. However, after 14 days, the temperature of the CS/PCL/BP scaffold increased by 11.4 °C after 10 min of irradiation, indicating a weakening of the photothermal performance. In comparison, the CS/PCL/BP/PDA scaffold showed a much better photothermal stability. The temperature rise of the CS/PCL/BP/PDA scaffold was 45.7 °C, which was close to the initial value of 51.2 °C after 14 days of exposure. Notably, the increase in the photothermal‐induced temperature (∆*T*) showed a slight decline under the protection of PDA after 14 days (89.3% of the ∆*T* of newly prepared scaffolds). In the meantime, the attenuation of temperature was remarkably distinct in the scaffold without the PDA layer (51.8% of the ∆*T* of the newly prepared scaffolds).

Furthermore, the stability of BP in the scaffolds was evaluated by determining the cumulative release curves of P from the CS/PCL/BP scaffolds with and without PDA coating after 7,14, and 28 days of water immersion (Figure 2c). Initially, the release amount of P maintained the same value (2.8 µg mL^−1^) for both CS/PCL/BP and CS/PCL/BP/PDA scaffolds after 7 days, whereas, the cumulative release amount of P from CS/PCL/BP scaffold reached 3.925 ± 0.035 µg mL^−1^ after 14 days and 5.08 ± 0.02 µg mL^−1^ after 28 days, which is significantly higher than that of the CS/PCL/BP/PDA scaffold (3.74 ± 0.05 µg mL^−1^ after 14 days and 4.25 ± 0.05 µg mL^−1^ after 28 days). Similarly, the cumulative release of P from the CS/PCL/BP and CS/PCL/BP/PDA scaffolds was consistent with the photostability results, further confirming the protective effect of PDA “envelope membrane”.

After the Ag nanoparticles were grown in situ into the scaffolds, the photothermal performances of the final CS/PCL/BP/PDA@Ag scaffolds with different initial Ag^+^ concentrations were studied (Figure 2d). Similar to the previous CS/PCL/BP/PDA scaffolds, the photothermal performance of chloroplast‐inspired CS/PCL/BP/PDA@Ag scaffolds still showed a concentration‐dependent behavior. With the addition of Ag nanoparticles, the photothermal performance of CS/PCL/BP/PDA@Ag scaffolds is better than that of CS/PCL/BP/PDA scaffolds. For instance, the temperature of the CS/PCL/BP/PDA@Ag scaffold with 6 mm initial AgNO_3_ concentration (CS/PCL/BP/PDA@Ag‐6 mm) increased significantly by 28.1 °C under 808 nm laser irradiation within 6 min (the initial temperature was 21.1 °C, power density was 0.5 W cm^−2^), whereas the temperature of CS/PCL and CS/PCL/PDA scaffolds increased by only 5.7 and 15.5 °C, respectively. Additionally, there was no significant attenuation of photothermal conversion in the CS/PCL/BP/PDA@Ag‐6 mm scaffold during five repeated cycles with the laser on and off, highlighting that the as‐designed scaffolds could be subjected to periodic NIR irradiation after implantation (Figure 2e). The infrared thermographic photographs visually revealed the strong hyperthermia of CS/PCL/BP/PDA@Ag scaffolds under NIR irradiation as compared to the CS/PCL scaffolds (Figure 2f). Taken together, the chloroplast‐inspired CS/PCL/BP/PDA@Ag scaffold possesses gratifying photothermal performance with durable stability.

### 2.3 Anti‐Infection Properties of the Chloroplast‐Inspired Scaffolds

**Figure 3:**
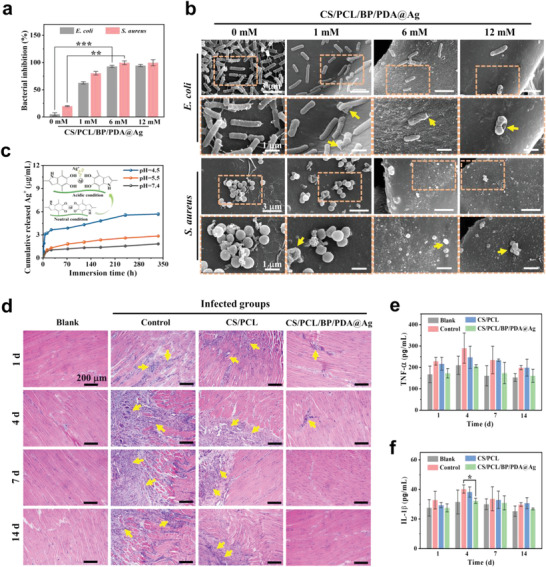
Evaluation of anti‐infection activities of chloroplast‐inspired self‐defensive scaffolds in vitro and in vivo. a) The bacterial inhibition ratio of gram‐negative *E. coli* and gram‐positive *S. aureus* cultured on the CS/PCL/BP/PDA@Ag scaffolds. b) SEM images of *E. coli* and *S. aureus* cultured on the CS/PCL/BP/PDA@Ag scaffolds under different magnification. The enlarged areas are marked with orange rectangular dashed line. Yellow arrows represent destroyed bacteria. c) Cumulative release curve of free Ag^+^ from CS/PCL/BP/PDA@Ag scaffold under different pH values (pH 7.4, 5.5, 4.5). Inset shows the possible schematic explanation of the pH‐triggered release behavior of Ag^+^ from scaffolds. d) Optical microscopy images of histopathological slices for blank (uninfected bone defect without any treatment), control (infected bone defect without any treatment), CS/PCL, and CS/PCL/BP/PDA@Ag groups via hematoxylin‐eosin (H&E) staining. Yellow arrows represent inflammatory cells. Release of inflammatory factors including e) rat tumor necrosis factor (TNF‐a) and f) interleukin 1*β* (IL‐1*β*) for blank, control, CS/PCL and CS/PCL/BP/PDA@Ag groups. Error bars: mean ± SD (*n* = 5), ^*^ *p* < 0.05, ^**^ *p* < 0.01, ^***^ *p* < 0.001.

Bacterial infections arising from orthopedic surgery or osteomyelitis remain a difficult clinical problem.^[^ ^3^ ^]^ Therefore, the bacterial inhibition ratio of CS/PCL/BP/PDA@Ag scaffolds against *E. coli* and *S. aureus* was investigated first in vitro. As shown in **Figure** **3**a, the antibacterial activity of the CS/PCL/BP/PDA@Ag scaffolds was positively correlated with the initial concentration of AgNO_3_. The bacterial inhibition ratios in CS/PCL/BP/PDA@Ag‐6 mm group (*S. aureus*: 99.5%, *E. coil*: 92.8%) and CS/PCL/BP/PDA@Ag‐12 mm group (*S. aureus*: 99.6%, *E. coil*: 95.1%) were high, whereas the bacterial inhibition ratio in the Ag‐free group was <20%. This result was further confirmed by the turbidity of bacterial suspensions co‐cultured with different scaffolds (Figure S7, Supporting Information). Meanwhile, the amount of *S. aureus* and *E. coli* adhered to CS/PCL/BP/PDA@Ag scaffolds significantly decreased with an increase in the initial Ag^+^ concentration. The cell membranes of *S. aureus* and *E. coli* cultured on PCL/BP/PDA@Ag scaffolds are obviously destroyed as denoted by the yellow arrows in Figure 3b. In contrast, *S. aureus* and *E. coli* were detected on CS/PCL/BP/PDA@Ag‐0 mm, and both showed intact morphology. Noteworthily, many bacterial pseudopodia appeared after culturing *E. coil* on CS/PCL/BP/PDA@Ag‐0, which is a symptom of bacterial biofilm formation. Moreover, the corresponding result of colony‐forming units (CFU) also illustrated the superior antibacterial properties of the CS/PCL/BP/PDA@Ag‐6 mm and 12 mm scaffold groups (Figure S8, Supporting Information).

The antibacterial effect of Ag nanoparticles is closely related to the release of Ag^+^, considering that free Ag^+^ can damage the normal function of bacterial proteins and further interfere with DNA replication.^[^ ^31^ ^]^ To verify this point, the CS/PCL/BP/PDA@Ag scaffolds were cultured with *S. aureus* and *E. coli* in agar plates for inhibition zone tests. A visible inhibition zone was observed in the CS/PCL/BP/PDA@Ag‐6 mm and 12 mm groups, indicating that the antibacterial property is closely related to Ag^+^ (Figure S9, Supporting Information). In general, bacterial infection can lead to a local acidic microenvironment.^[^ ^32^ ^]^ Therefore, the release behavior of Ag^+^ in the CS/PCL/BP/PDA@Ag scaffold under different pH values (pH 7.4, 5.5, 4.5) was studied as well. As shown in Figure 3c, there was a significant variation in Ag^+^ release when the pH values were changed from physiological microenvironment (pH 7.4) to an affected acidic microenvironment (pH 5.5, 4.5), exhibiting a pH‐responsive release behavior of Ag^+^ in the CS/PCL/BP/PDA@Ag scaffold. Comparatively, the cumulative amount and release rate of Ag^+^ were much higher at pH 4.5 than that at pH 5.5, and the lowest at pH 7.4, indicating that Ag^+^ liberation is accelerated from the CS/PCL/BP/PDA@Ag scaffold under a bacteria‐triggered acidic metabolic microenvironment. As illustrated in the inset, the in situ anchoring strategy enhances the interfacial bonding force between the Ag nanoparticles and PDA by Ag‐phenolic oxy‐group coordination.^[^ ^33^ ^]^ Under local acidic conditions, the Ag‐O coordination is broken, and the Ag‐phenolic complex is converted to phenolic hydroxyl. Subsequently, Ag^+^ ions quickly eluted from the CS/PCL/BP/PDA@Ag scaffold to kill the bacteria.^[^ ^34^ ^]^ Based on this, the CS/PCL/BP/PDA@Ag scaffold was endowed with self‐adaptive antibacterial characteristics triggered by the metabolism of bacteria. Particularly, a responsive antibacterial process could be intelligently implemented depending on the degree of infection, which mimics the self‐defensive functionality of chloroplasts well. Simultaneously, cellular assays of the chloroplast‐inspired CS/PCL/BP/PDA@Ag scaffolds in vitro also demonstrated satisfactory cytocompatibility (Figure S10, Supporting Information). Based on the adequate photothermal properties, satisfactory antibacterial ability, and good cytocompatibility, the CS/PCL/BP/PDA@Ag scaffold with 6 mm initial Ag^+^ concentration was selected for subsequent experiments.

We then evaluated the ability of CS/PCL/BP/PDA@Ag scaffolds to fight against bacteria‐associated infections in vivo. To this end, critical‐size femoral defects with a diameter of 2.5 mm and depth of 3 mm were infected with *S. aureus* to establish an implant‐related infection in SD rats (Figure S11a, Supporting Information). The as‐designed CS/PCL/BP/PDA@Ag scaffold was then implanted into the femoral defects with bacterial infection for 1, 4, 7, and 14 days, along with CS/PCL scaffold, blank (pure defects without injection of bacterium suspension), and control (pure defects with injection of bacterial suspension) groups for comparison. The corresponding tissue slices were analyzed by H&E staining (Figure 3d). Compared with the blank group, there were many inflammatory cells in the control and CS/PCL groups over the entire experimental period (yellow arrows represent inflammatory cells), indicating a large area of infected tissues. In contrast, inflammatory cells peak after 4 days in the CS/PCL/BP/PDA@Ag group and then decrease gradually with no inflammatory cells can be observed after 14 days, demonstrating that the infection was effectively inhibited upon implantation of the chloroplast‐inspired CS/PCL/BP/PDA@Ag scaffold.

In addition, the related inflammatory factors were quantitatively determined using an ELISA kit. As the inflammatory cytokines related to bacterial infection, the concentration of TNF‐*α* and IL‐1*β* in all groups increased from 0 day to 4 days, and then decreased gradually (Figure 3e,f). During the initial 4 days, high expression of inflammatory cytokines TNF‐*α* and IL‐1*β* indicated that the high bioactivity of macrophages was triggered by bacterial infection and exogenous material implantation. Subsequently, the inflammatory response reduced gradually due to the eradication of bacteria, especially in the CS/PCL/BP/PDA@Ag group. In addition, IL‐6 cytokine, a pro‐inflammatory and anti‐inflammatory cytokine secreted by macrophages, exhibited a slight increase in the blank and CS/PCL/BP/PDA@Ag groups after 14 days of implantation, whereas that of the control and CS/PCL groups decreased gradually after 7 days (Figure S11b, Supporting Information). The high expression of IL‐6 in the blank and CS/PCL/BP/PDA@Ag groups can be ascribed to pro‐inflammatory and anti‐inflammatory effects throughout the study period.^[^ ^35^ ^]^ All cytokines detected in the CS/PCL/BP/PDA@Ag group were similar with those in the blank group, and the positive outcomes confirmed the excellent anti‐infection property of the chloroplast‐inspired CS/PCL/BP/PDA@Ag scaffold in vivo.

### 2.4 In Vitro Studies of Osteogenesis

**Figure 4:**
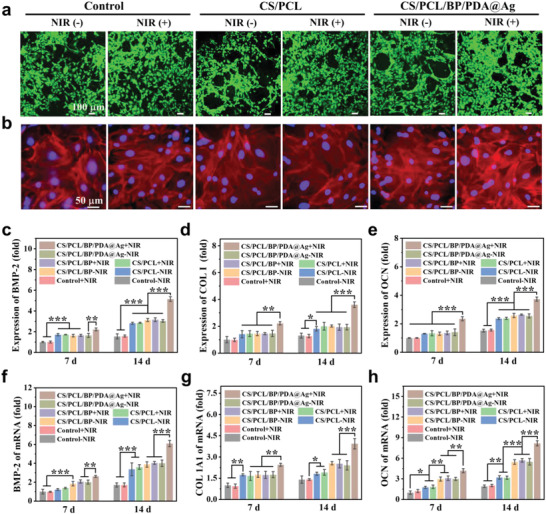
Assessments of cell viability and osteogenesis in vitro. a) Live/dead staining and b) fluorescence microscopy images of rBMSCs cells incubated with the control (cells without scaffold), CS/PCL, and CS/PCL/BP/PDA@Ag scaffolds for 3 days stimulated with or without NIR irradiation (scale bars = 100 and 50 µm, respectively), green and red in figure (a) represent living cells and dead cells, respectively. F‐actin and nucleus of cells in figure (b) is stained in red and blue, respectively. The expressions of osteogenic c–e) proteins and f–h) genes in rBMSCs after osteo‐induction for 7 and 14 days (taking control – NIR group as reference). Error bars: mean ± SD (*n* = 5), ^*^ *p* < 0.05, ^**^ *p* < 0.01, ^***^ *p* < 0.001.

To investigate the effect of BP and NIR‐induced hyperthermia on cell function, rBMSCs were incubated on different scaffolds for the assessment of viability and osteogenic differentiation. Mild hyperthermia (40–42 °C) has been demonstrated favorable for bone regeneration,^[^ ^36^ ^]^ and this temperature range was applied in this study. It was clearly observed that negligible dead cells were observed on all scaffolds (**Figure** **4**a). Meanwhile, the cells in all groups exhibited spindle‐like morphology, whose nucleus and cytoskeleton were clearly identified (Figure 4b). As time extended, the relative cellular proliferation ratio in all groups increased, especially for CS/PCL/BP/PDA@Ag + NIR group (Figure S12, Supporting Information), which could significantly facilitate growth and proliferation of rBMSCs.

Besides, the osteogenesis‐associated protein and gene expressions of rBMSCs were measured under periodic photothermal stimulation. The cell experiments were divided into eight groups, namely control ± NIR, CS/PCL ± NIR, CS/PCL/BP ± NIR, and CS/PCL/BP/PDA@Ag ± NIR groups (“‐” and “+” represents without and with NIR irradiation, respectively). Collectively, the expression of the four kinds of osteogenic proteins and genes including bone morphogenetic protein 2 (BMP‐2), type I collagen (Col I), osteocalcin (OCN), and alkaline phosphatase (ALP) showed the similar variation at both 7 and 14 days (Figure 4c–h; Figure S13, Supporting Information). Notably, the relative expression values of both proteins and genes in CS/PCL/BP/PDA@Ag + NIR group kept the highest, whereas the values in other scaffold groups were lower and showed no much difference with or without NIR stimulation, indicating that CS/PCL/BP/PDA@Ag scaffolds under mild hyperthermia could significantly promote osteogenic differentiation. Meanwhile, the relative gene expression of OCN in BP‐based groups exhibited an increase in contrast to the CS/PCL groups at 14 days, also proving the osteogenic effect of BP nanosheets.

**Figure 5:**
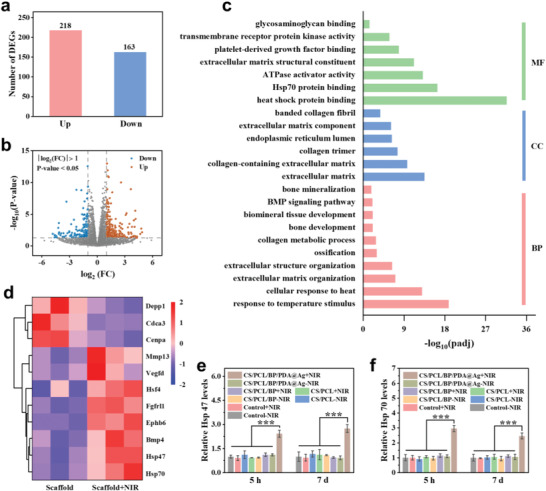
Transcriptome sequencing analysis of the mechanism of NIR stimulation for osteogenesis. a) The number of upregulated and downregulated genes. b) The volcano plot of transcriptomic results of 21 323 DEGs in scaffold + NIR group compared with the scaffold group. c) GO analysis including BP, CC, and MF. d) Hot map of DEGs related to hyperthermia and osteogenesis between scaffold + NIR group and scaffold group (*n* = 3). RT‐PCR detection of the e) Hsp 47 and f) Hsp 70 expression levels in rBMSCs treated with scaffolds and NIR irradiation for 5 h and 7 days. Error bars: mean ± SD (*n* = 5), ^***^ *p* < 0.001.

On the basis of the above study, bioinformatics analysis of RNA transcriptome sequencing (RNA‐seq) was performed to explore the osteogenic mechanism of photothermal stimulation. Here, rBMSCs were co‐cultured with CS/PCL/BP/PDA@Ag scaffold with/without NIR irradiation (defined as scaffold+ NIR and scaffold, respectively), and total RNA of cells was extracted. Differential expression genes (DEGs) analysis (**Figure** **5**a) and volcano plot (Figure 5b) revealed that 218 genes were upregulated and 163 genes were downregulated in scaffold+NIR group.

GO analysis of the RNA‐seq revealed that scaffold + NIR treatment altered the biological process (BP), cellular component (CC), and molecular function (MF). Most of changes were significantly involved in hyperthermia and osteogenesis, such as the cellular response to heat, extracellular matrix, heat shock protein binding, Hsp 70 protein binding and ATPase activator activity (Figure 5c). Meanwhile, KEGG enrichment analyses of DEGs in rBMSCs illustrated that many pathways associated with osteogenesis including PI3K‐Akt, signaling pathways regulating pluripotency of stem cells, MAPK and Rap1 were regulated in scaffold+ NIR group (Figure S14, Supporting Information). Consistently, the hot map visualized the significant upregulation of the heat shock protein/factor (such as Hsp 70 and Hsp 47) and osteogenesis‐related genes (such as Bmp 4, Mmp 13, and Vegfd) in rBMSCs under NIR irradiation (Figure 5d).

Previous research proved that the heat shock proteins of Hsp 47 (related to the maturation of osteogenesis‐related collagen I molecules) and Hsp 70 (associated with the thermo‐tolerance of osteoblasts) were closely related to osteogenesis.^[^ ^9^, ^37^ ^]^ To further clarify the osteogenic effect of photothermal stimulation, hence, the expressed levels of Hsp 47 and Hsp 70 in both instantaneous (5 hours) and persistent (7 days) hyperthermal stress under NIR irradiation were verified by RT‐PCR (Figure 5e,f). The relative gene levels of Hsp 47 and Hsp 70 were significantly upregulated in both 5 h and 7 days in the CS/PCL/BP/PDA@Ag + NIR group, while the gene expressions in other groups were insensitive. Consistent with RNA‐seq results, CS/PCL/BP/PDA@Ag scaffold was capable of osteogenesis by upregulating the heat shock‐related genes under mild photothermal stimulation. To sum up, CS/PCL/BP/PDA@Ag scaffold with mild photothermal stimulation could significantly increase the expression of osteogenesis‐related genes, proteins, and pathways, and thus involved in the osteogenic differentiation and bone metabolism.

### 2.5 In Vivo Bone Regeneration of the Chloroplast‐Inspired Scaffolds under Infected Condition

The superior anti‐infection properties of bioinspired scaffolds had been confirmed in vitro and in vivo, respectively. The potential of NIR‐mediated photothermal osteogenesis for chloroplast‐inspired scaffolds was investigated further using an infected femoral defect rat model. In this section, rats were randomly assigned to four groups: the CS/PCL ± NIR and CS/PCL/BP/PDA@Ag ± NIR groups (“‐” and “+” represent without and with NIR irradiation, respectively). Similar to the photothermal results in vitro, the photothermal performance of the CS/PCL/BP/PDA@Ag scaffolds also showed power density‐dependent characteristics in vivo (Figure S15, Supporting Information). Based on the studies in vitro, the laser power density of 0.75 W cm^−2^ was used to study the photothermal osteogenesis in vivo because its hyperthermal temperature range is satisfactory.

**Figure 6:**
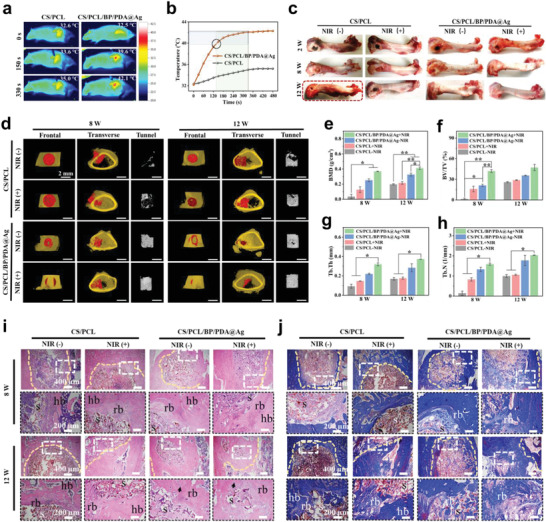
In vivo bone regeneration of femoral defects with bacterial infection after 8 and 12 weeks of implantation with or without periodical NIR irradiation. a) Infrared thermographic maps of the highest temperature in irradiated areas. b) Time‐dependent temperature curve of the CS/PCL and CS/PCL/BP/PDA@Ag implants in vivo irradiated with the 808 nm laser (0.75 W cm^−2^). c) Photographs of femoral tissues after 2, 8, and 12 weeks of implantation. d) Frontal and transverse views of the 3D‐reconstructed images with micro computed tomography (micro‐CT) analysis (where the defected areas are highlighted with red color). Characteristic 3D micro‐CT images within a region of interest of the central 2.5 mm diameter of the bone tunnel at 8 and 12 weeks after surgery. e) Quantitative analysis of bone mineral density (BMD), f) bone volume/tissue volume (BV/TV), g) trabecular thickness (Tb, Th), and h) trabecular number (Tb. N) in defect sites. i) H&E staining and j) Masson trichrome staining of the transplanted sites under different magnification. Meanwhile, hb, host bone. rb, regenerated bone. s, scaffold. Black arrow, bone marrow space. Orange dashed line, demarcation between host and regenerated bones. The enlarged areas are marked with rectangular dashed line. Error bars: mean ± SD (*n* = 5), ^*^ *p* < 0.05, ^**^ *p* < 0.01.

The images of the infrared thermograph in lesion locations were recorded, as shown in **Figure** **6**a. The temperature of the CS/PCL group increased only from 32.6 to 35.6 °C in 330 s, while the CS/PCL/BP/PDA@Ag group showed a significant temperature increase from 32.6 to 39.6 °C after 150 s, and to 42.1 °C after 330 s. This temperature remained unchanged under further irradiation, indicating that stable heat exchange was achieved at this time point (Figure 6b). It is also a direct indicator that NIR light can penetrate the skin tissue and trigger CS/PCL/BP/PDA@Ag scaffold heating in vivo.

After 2, 8, and 12 weeks of implantation, the femurs were harvested from the rats. The defects in the CS/PCL groups with or without NIR irradiation were obviously larger than those in the CS/PCL/BP/PDA@Ag groups at any preset time and did not repair significantly over time (Figure 6c). As expected, the repair effect of CS/PCL/BP/PDA@Ag + NIR group was significantly better than that of the other groups with regard to the near disappearance of defects after 12 weeks of implantation. In contrast, the defects in the CS/PCL – NIR group had been developed to osteomyelitis due to serious bacterial infection after 12 weeks of implantation (highlighted by the red rectangles). Besides, at 8 and 12 weeks, heavy festering happened owing to the appearance of swelling and pustule (highlighted by white dashed circles) in the CS/PCL ± NIR groups, while no apparent symptom was found in the CS/PCL/BP/PDA@Ag ± NIR groups (Figure S16, Supporting Information). These phenomena, in turn, further proves that the CS/PCL/BP/PDA@Ag scaffolds simultaneously show the anti‐infection and osteogenic properties in vivo.

To visualize osteogenesis, frontal and transverse microstructures of bone defects were reconstructed by micro‐CT imaging as shown in Figure 6d (where the unrepaired defect areas are highlighted in red). Overall, the repair effect was time‐dependent in all the groups. Owing to the absence of Ag nanoparticles in the CS/PCL ± NIR groups, the repair effect was worse than that of the CS/PCL/BP/PDA@Ag ± NIR groups, signifying that the robust self‐defensive property of implants is a prerequisite for bone defects healing under infected condition. Moreover, the synergistic positive effect of BP and PDA was expected to be beneficial for interfacial bioactivity and osteo‐inductivity, as evidenced by the excellent biomineralization properties of the CS/PCL/BP/PDA@Ag scaffolds (Figure 1i). Under NIR irradiation, more fresh bone tissues were regenerated in the CS/PCL/BP/PDA@Ag + NIR group than that in the CS/PCL/BP/PDA@Ag – NIR group, corroborating that the moderate photothermal stimulation is favorable to bone regeneration. The corresponding 3D reconstructed images of the bone defect tunnel also verified these results well. Noteworthily, the volume of new bone was the highest in the CS/PCL/BP/PDA@Ag + NIR group.

In addition, the parameters of osteogenesis, including BMD, BV/TV, Tb. Th, and local Tb. N in all the groups showed a similar trend, as described above (Figure 6e–h). From 8 to 12 weeks, these parameters of osteogenesis in the CS/PCL/BP/PDA@Ag + NIR group had the highest values, followed by those in the CS/PCL/BP/PDA@Ag – NIR group, and the CS/PCL group had the lowest values. Typically, after 12 weeks of implantation, the values of BMD, BV/TV, Tb. Th, and Tb. N in the CS/PCL/BP/PDA@Ag + NIR group were 0.41 g cm^−3^, 47.15%, 0.37 mm, and 2.03 mm^−1^, respectively, which were higher than those of the CS/PCL/BP/PDA@Ag – NIR group (0.326 g cm^−3^, 35.55%, 0.28 mm, and 1.77 mm^−1^), CS/PCL + NIR group (0.216 g cm^−3^, 28.88%, 0.18 mm, and 1.06 mm^−1^), and CS/PCL – NIR group (0.19 g cm^−3^, 25.89%, 0.16 mm, and 0.99 mm^−1^). Evidently, the chloroplast‐inspired CS/PCL/BP/PDA@Ag scaffold could effectively suppress the infection at an early stage of implantation and promote new bone formation thereafter. In particular, the bone repair effect of CS/PCL/BP/PDA@Ag scaffold can be further enhanced upon mild NIR irradiation.

For the histological assessment of newly formed bone in defects, H&E and Masson staining were performed to trace the process of bone formation in vivo. As shown in Figure 6i, some residual inflammation‐associated cells were still found in the CS/PCL ± NIR groups after 8 weeks of implantation, verifying that the CS/PCL scaffold was not resistant to bacteria. Thus, the defect recovery process in the two CS/PCL groups was somewhat delayed. Conversely, the inflammatory cells were hardly found in the CS/PCL/BP/PDA@Ag ± NIR groups because of the sustained antibacterial ability of Ag^+^ release. Consequently, the areas of new bone formation in the two CS/PCL/BP/PDA@Ag groups were larger than those in the CS/PCL groups at the same time points. Particularly in the CS/PCL/BP/PDA@Ag + NIR group, the fresh osseous tissue exhibited a compact and integrated structure. Furthermore, bone marrow space (shown by black arrows) could be detected, suggesting that new bone tissue turns to maturity.^[^ ^38^ ^]^

Furthermore, Masson staining (blue represents type I collagen) (Figure 6j) revealed that the regenerated bone (rb) and collagen fibrous tissues growing from the surrounding host bone (hb) tissue were integrated and intertwined with the scaffolds, as indicated by the orange dashed line. The defect areas in the CS/PCL/BP/PDA@Ag + NIR group were smaller than those in the non‐irradiated group, implying that the growth of new bones could be accelerated by effective photothermal stimulation. Synchronously, loose collagen fibers integrated into the CS/PCL/BP/PDA@Ag scaffolds became denser and thicker from 8 to 12 weeks of implantation. These results corroborate that the CS/PCL scaffold modified with PDA and BP layers can promote the growth and maturity of new bone matrix, while the process is further accelerated by suitable photothermal stimulation. Therefore, the chloroplast‐inspired CS/PCL/BP/PDA@Ag scaffold provides the excellent anti‐infection ability and photothermal osteogenesis potential in the case of infected bone defects, thus achieving the effect of killing two birds with one stone.

**Figure 7:**
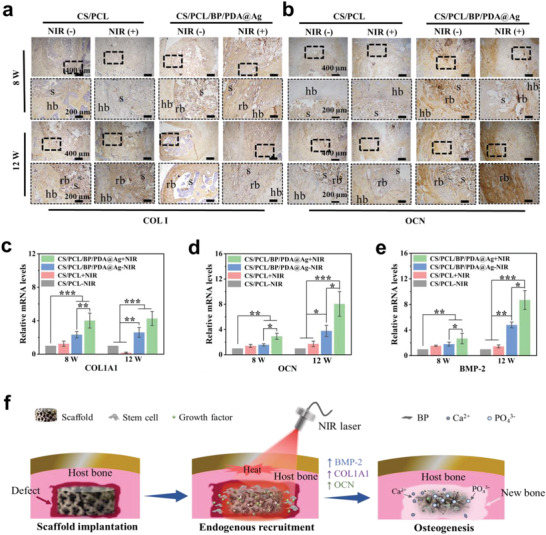
Immunohistochemical staining and gene expression of peri‐implant tissues after 8 and 12 weeks of implantation with or without periodic NIR irradiation. a,b) Positive brown staining of COL I and OCN productions under different magnification. hb, host bone. rb, regenerated bone. s, scaffold. The enlarged areas are marked with rectangular dashed line. The gene expression of c) COL1A1, d) OCN, and e) BMP‐2 analyzed by RT‐PCR. f) Possible mechanism of osteogenesis with the combination of heat stimulation and BP‐based platform. Error bars: mean ± SD (*n* = 3), ^*^ *p* < 0.05, ^**^ *p* < 0.01, ^***^ *p* < 0.001.

To further investigate bone repair at the molecular and genetic levels, bone‐specific proteins and osteogenesis‐related gene expression of the peri‐implant tissues. The protein expression of COL I and OCN (**Figure** **7**a,b) was analyzed by immunohistochemical staining. The distinct brown region indicated positive expression of COL I and OCN in regenerated bone (rb) areas, which was differentiated with that of host bone (hb). Notably, the positive expression levels of COL I and OCN in CS/PCL/BP/PDA@Ag + NIR group are much higher than those of the other groups accompanied by a reduction in defect areas. Similarly, the osteo‐inductive protein expression of BMP‐2 was consistent with the above results (Figure S17, Supporting Information).

Finally, the osteogenesis‐related genes of COL1A1, OCN, and BMP‐2 (Figure 7c–e) in newly regenerated tissue were quantitatively determined by RT‐PCR. The relative gene expressions rank at the order of CS/PCL/BP/PDA@Ag + NIR group > CS/PCL/BP/PDA@Ag – NIR group > CS/PCL + NIR group. Specifically, the gene expression levels of COL1A1, OCN and BMP‐2 in chloroplast‐inspired scaffolds with NIR irradiation were 1.64, 2.13, and 1.81 folds higher than that of without NIR irradiation at 12 weeks of implantation, respectively. Therefore, these results indicate that the CS/PCL/BP/PDA@Ag scaffold can facilitate infected bone repair, especially upregulating the expression of osteogenesis‐related genes under periodic NIR irradiation.

The possible mechanism of osteogenesis for chloroplast‐inspired scaffolds mainly originates from two points. For one thing, this may be due to the fact that mild thermal stimulation could facilitate the expression of heat shock proteins and finally mediate osteogenesis in vivo, as discussed in Figure 5. For another, abundant endogenous stem cells and growth factors in host bone tissue could be recruited to defect areas with the bone‐like hierarchical structure and bioactive ingredients of BP and PDA in the CS/PCL/BP/PDA@Ag scaffold, providing a favorable microenvironment for osteogenic differentiation (Figure 7f).^[^ ^28^ ^]^ Following the collapse of the scaffold in vivo, especially the degradation of BP nanosheets therein, many PO_4_^3−^ are released as the precursor ions of bone inorganic ingredients, and thus capture Ca^2+^ from physiological fluid to generate inorganic minerals in newborn osseous tissue beneficial for osteogenesis. Furthermore, osteogenesis‐related signaling pathways, including COL1A1, BMP‐2, and OCN, might be activated synergistically through mild thermal stimulation. Taken together, all of the tests in vivo prove that the chloroplast‐inspired scaffolds promote osteogenesis under infective conditions with a facile external control of NIR irradiation. This chloroplast‐inspired strategy provides a practical and effective platform for bone repairing.

## 3 Conclusion

In summary, by studying the unique light conversion and defense protection properties of chloroplasts, we successfully designed a novel CS/PCL/BP/PDA@Ag scaffold with prominent photothermal osteogenesis and self‐defensive ability for infected bone defect therapy. The biocompatible scaffold provided a favorable microenvironment for cell growth with a bone‐like 3D porous structure, outstanding biomineralization ability, suitable degradation rate, and water uptake/retention ratio. Particularly, the chloroplast‐inspired platform shows satisfying photothermal stability owing to the protection of the PDA “envelope membrane”, and hence, the incremental ratio of photothermal‐induced temperature in the PDA‐protected scaffold (89.3%) was higher than that of the non‐protected scaffold (51.8%) until 14 days of testing. In situ anchoring of Ag nanoparticles endowed scaffolds with robust antimicrobial ability and anti‐infection potential in vitro and in vivo on account of the rapid responsive release of free Ag^+^ in the acidic microenvironment of bacterial infection. Furthermore, bacteria‐triggered infection can be completely inhibited by chloroplast‐inspired scaffold at an early stage and effectively promote endogenous repair of femur defects under mild NIR irradiation thereafter.

## 4 Experimental Section

Detailed experimental materials and methods can be found in the Supporting Information. All animal studies were performed with the permission of the Animal Research Committee of Sichuan University (approval number: 2019065A).

## Conflict of Interest

The authors declare no conflict of interest.

## Supporting information

Supporting Information

## Data Availability

Research data are not shared.
