# Supplementary material for: Chloroplast‐inspired Scaffold for Infected Bone Defect Therapy: Towards Stable Photothermal Properties and Self‐Defensive Functionality
Source: Adv Sci (Weinh). 2022 Sep 15;9(31):2204535. doi: 10.1002/advs.202204535 (PMC9631053; doi:10.1002/advs.202204535)
Supplement: Supplementary file 1 — Supporting Information [file ADVS-9-2204535-s001.pdf]

ADVANCED SCIENCE
Open Access

Supporting Information

for *Adv. Sci.*, DOI 10.1002/advs.202204535

Chloroplast-inspired Scaffold for Infected Bone Defect Therapy: Towards Stable Photothermal Properties and Self-Defensive Functionality

Yao Zhao, Xu Peng, Dingqian Wang, Hongbo Zhang, Qiangwei Xin, Mingzhen Wu, Xiaoyang Xu, Fan Sun, Zeyuan Xing, Luning Wang, Peng Yu, Jing Xie, Jiehua Li, Hong Tan, Chunmei Ding and Jianshu Li**

Supporting Information

Chloroplast-inspired scaffold for infected bone defect therapy: towards stable photothermal properties and self-defensive functionality

Yao Zhao, Xu Peng, Dingqian Wang, Hongbo Zhang, Qiangwei Xin, Mingzhen Wu, Xiaoyang Xu, Fan Sun, Zeyuan Xing, Luning Wang, Peng Yu, Jing Xie, Jiehua Li, Hong Tan, Chunmei Ding, Jianshu Li **

Dr. Y. Zhao, Prof. X. Peng, D. Wang, H. Zhang, Dr. Q. Xin, M. Wu, X. Xu, F. Sun, Z. Xing, L. Wang, P. Yu, Prof. J. Xie, Prof. J. Li, Prof. H. Tan, Prof. C. Ding, Prof. J. Li
College of Polymer Science and Engineering, State Key Laboratory of Polymer Materials Engineering, Sichuan University, Chengdu 610065, China
E-mail: jianshu_li@scu.edu.cn; dingcm@scu.edu.cn

Prof. X. Peng
Experimental and Research Animal Institute, Sichuan University, Chengdu 610065, China

Prof. J. Li
State Key Laboratory of Oral Diseases, West China Hospital of Stomatology, Med-X Center for Materials, Sichuan University, Chengdu 610041, China

Contents:

1. Supplementary Methods (1.1-1.18)

2. Supplementary Figures (Figure S1-S17)

3. Supplementary Table (Table S1 and S2)

1. Supplementary Methods

1.1. Materials.

Chitosan (CS, 90% deacetylated) and Poly-ε-caprolactone (PCL, MW: 80,000) were purchased from Shanghai yuanye Bio-Technology Co., Ltd (Shanghai, China). Bulk BP crystals (> 99.999%) were purchased from Nanjing MKNANO Tech. Co., Ltd (Nanjing, China). Silver nitrate ($AgNO_3$) (99.8%) was obtained from Haihong Chemical Company (Chengdu, China). Dopamine hydrochloride (DA) was obtained from Solarbio (Beijing, China). Fetal Bovine Serum (FBS), Alpha Modified Eagle Medium (α-MEM) and other reagents for cell culture were purchased from Bosco Biotechnology Co., Ltd (Chengdu, China). All of the chemicals were of analytical grade (A.R.). Deionized water (15.0 MΩ. cm) was used in all experiments.

1.2. Synthesis of BP nanosheets.

A liquid exfoliation method was employed to prepare BP nanosheets.[1] Briefly, the bulk BP crystals (15 mg) were ground into powder and dispersed in N-methyl-2-pyrrolidone (NMP) (30 mL). Then, the mixture was sonicated in an ice bath at the power of 300 W for 10 hours. The solution was centrifuged at 4000 rpm for 10 minutes to remove the non-exfoliated BPs and then supernatant was gently collected for later use. The lateral size of BP nanosheets were counted by Image J statistical analysis software.

1.3. Fabrication of chloroplast-inspired CS/PCL/BP/PDA@Ag scaffolds.

For the CS/PCL/BP/PDA@Ag scaffold, CS scaffold was firstly prepared by vacuum freeze-drying technology. Then, CS scaffold was mechanically enhanced with PCL polymer, followed by the functionalization with carboxyl groups. After that BP nanosheets were integrated into CS/PCL scaffold and stabilized by PDA. Afterwards, Ag nanoparticles were *in situ* anchored into scaffold, and thus the chloroplast-inspired CS/PCL/BP/PDA@Ag scaffold was obtained. The details are described as follow.

Firstly, CS was added into acetic acid (2%, w/v) for complete dissolution under the condition of 37 °C water bath by magnetic stirring. CS solution was then injected into the cylindrical mold

and frozen overnight in the refrigerator. The next day, it is freeze-dried in the freeze-dryer. Secondly, PCL granules were added into the mixed solution of dichloromethane and ethanol (4:1, v/v) and stirred until they were completely dissolved. Then, acquired PCL solution was infiltrated into the 3D porous CS scaffolds with constant volume. The CS/PCL (Unt) scaffolds were obtained by solvent evaporation at room temperature. Whereafter, the CS/PCL (Unt) scaffolds were immersed into 1M NaOH solution in a shaker incubator (37 °C). After 1 hour, the NaOH treated CS/PCL scaffolds were removed, carefully washed with deionized water and dried at room temperature. The obtained CS/PCL scaffolds above were soaked in BP ethanol solution (50, 100, and 150 ppm) for 10 minutes, and then dried again at room temperature. This procedure was repeated three times to obtain the CS/PCL/BP scaffolds. The surface modification with poly(dopamine) was performed by immersing CS/PCL/BP scaffolds into a dopamine solution (2 mg/mL in 10 mM Tris, pH 8.5) at 25 °C for 24 hours. Subsequently, the modified scaffolds were washed with water and then dried with nitrogen gas. Finally, the CS/PCL/BP/PDA scaffolds were immersed in $AgNO_3$ solution (1, 6 and 12 mM), followed by continuous magnetic stirring for 3 hours in darkness. After that, the scaffolds were washed for several times to remove the unreacted Ag^+ on the surface and freeze-dried again to obtain CS/PCL/BP/PDA@Ag multifunctional scaffolds. The CS/PCL/BP/PDA@Ag scaffolds with different initial $AgNO_3$ concentrations (1, 6 and 12 mM) are defined as CS/PCL/BP/PDA@Ag-1mM, CS/PCL/BP/PDA@Ag-6mM and CS/PCL/BP/PDA@Ag-12mM.

1.4. Physicochemical characterization of the chloroplast-inspired scaffolds.

The scanning electron microscopy (SEM, Apreo S HiVoc, USA) images and element mapping were obtained using a Nova Nanosem 430 scanning electron microscope at an acceleration voltage of 15 kV. The crystalline structure of each sample was analyzed by X-ray diffraction (XRD, Ultima IV, Japan). Inductively coupled plasma optical emission spectrometry (ICP-OES, Optima 7000DV, PerkinElmer, USA) was applied to measure the concentration of Ag^+. Fourier transform infrared spectroscopy (FTIR; Nicolet 6700, Thermo Scientific TM, USA) with the

measuring range of 4000 to 400 cm^{-1} was employed to detect organic components of the scaffold. Meanwhile, Raman scattering was carried out on a Lab Ram HR visible high-resolution confocal Raman microscope equipped with a 633 nm laser. The junction state of the elements in scaffolds were performed using an X-ray photoelectron spectroscopy (XPS, Axis Supra, UK). To evaluate the photothermal performance of the samples, an 808 nm NIR laser equipped with an infrared thermal imaging camera (Testo 885-2, Germany) was employed.

1.5. Porosity and swelling ratio measurement of the chloroplast-inspired scaffold.

A liquid displacement method was used to assess the porosity of scaffolds.[2] Briefly, the dried scaffold with a mass of W_d was immersed in a test tube which has enough ethanol in order to submerge the sample for 5 minutes. The test tube was transferred to a glass desiccator under vacuum condition so as to compel the ethanol into the gap of the scaffolds. When ethanol was entirely removed from the surface, the ethanol infiltrated scaffold was weighted (W_w). Each measurement was repeated 5 times. The porosity of the scaffold was tested by the following formula.

Porosity (%) = $(W_w - W_d) / (\rho\times\pi\times h\times(d/2)^2)\times 100$ (1)

Where ρ represents the density of ethanol (0.789 g/cm^3). π is the pi value 3.14. d is the diameter and the h is the thickness of the scaffold.

Moreover, scaffolds with equivalent weights were soaked in phosphate-buffered saline solution (PBS) at 37 °C to detect swelling ratio with different time. Briefly, scaffolds with equal weights were immersed in 15 mL PBS at 37 °C. At predetermined intervals (12, 24, 48, 72 and 120 hours), the scaffolds were taken out. The excess PBS was removed with filter paper and the weight of the scaffolds was measured. The swelling ratio was calculated using the equation as follow.

Swelling ratio (%) = $(M_2 - M_1) / M_1\times 100$ (2)

Where the M_1 and M_2 are the weights of the samples before and after immersing in PBS.

1.6. The water uptake and retention rates of the chloroplast-inspired scaffold.

Dry scaffolds were weighed (W_d), immersed in distilled water for 48 hours, blotted with filter paper, and weighed again (W_{w1}) to determine the water uptake rate. Water retention rates were estimated by centrifuging the wet scaffolds (500 rpm, 3 min), followed by weighing (W_{w2}). The percent equilibria of water uptake and water retention were calculated using equations (3) and (4).

Water uptake ratio (%) = $(M_{w1} – M_d) / M_d \times 100$ (3)

Water retention ratio (%) = $(M_{w2} – M_d) / M_d \times 100$ (4)

1.7. Analysis of mechanical properties of the chloroplast-inspired scaffold.

The universal material testing machine (INSTRON model 1185, Shimadzu) at a cross-head speed of 1.0 mm/min was utilized to detect mechanical properties of the scaffolds (Φ15×4 mm). The compressive stress was recorded with the deformation of 65%. Three parallel samples were used during the measurement.

1.8. *In vitro* degradation of the chloroplast-inspired scaffold.

The degradation behavior of the composite scaffolds was tested by immersing the samples in PBS with lysozyme (0.5 mg/mL) in a shaking bath at 37°C. The solution was refreshed every two days. At a preset time point, the scaffolds were cleaned in order to wipe off the PBS residual. After that, the scaffolds were lyophilized and weighted. Five parallel samples were taken into account for each group. The weight loss ratio was determined by the equation.

Weight loss ratio (%) = $(W_2 - W_1) / W_2 \times 100$ (5)

Where W_1 and W_2 are the initial and final weight after incubation of the composite scaffolds, respectively.

1.9. Ag^+ release from the chloroplast-inspired CS/PCL/BP/PDA@Ag scaffold in distinct pH of PBS.

Release behavior of Ag^+ from the CS/PCL/BP/PDA@Ag scaffold was studied. Briefly,

specimens were immersed in PBS (10 mL) with distinct pH values (pH = 7.4, 5.5, 4.5) at 37 °C for consecutive 14 days in a shaking bath. At the preplanned time (1 h, 3 h, 6 h, 12 h, 1 d, 3 d, 5 d, 7 d, 10 d, 14 d), above solution was collected for determining the concentration of Ag^+ and then an equal volume of fresh deionized water was added. The cumulative release concentrations of Ag^+ were detected by ICP-OES.

1.10. *In vitro* biomineralization study of the chloroplast-inspired scaffold.

The scaffolds were immersed in test tube with 30 mL of simulated body fluid (SBF) at 37 °C for 1 and 5 days. The solution was refreshed every two days, and the biomineralized scaffolds were studied by SEM and XRD.

1.11. Cytocompatibility study of prepared scaffold with different BP and initial Ag^+ concentrations.

Mouse pre-osteoblast MC3T3-E1 sub-clone cells were used for the appraisal of cytocompatibility. The MC3T3-E1 cells were cultured in complete a-MEM medium, which contains 100 U/mL penicillin, 0.1 mg/mL streptomycin and 10% FBS. The cells were incubated in a CO_2 incubator (MCO-15AC, SANYO, Japan) at 37 °C under humid conditions in the presence of 5% CO_2. Cells in the logarithmic phase of growth were used for further studies. Additionally, dead (red) and live (green) cells in 1 and 3 days were stained by Fluorescein Diacetate and Propidium Iodide (FDA/PI) blend dyes, and observed using a fluorescence inverted microscope (OLYMPUS, Japan). In addition, the cells were fixed with 4% paraformaldehyde, permeabilized with 0.1% Trion X-100 in PBS, and F-actin and nucleus of cells were stained with Rhodamine-labeled Phalloidin (red) and 4', 6-Diamidino-2-Phenylindole (DAPI, blue), respectively. The proliferation of cells cultured with extracting liquid of scaffolds was investigated by Cell Counting Kit-8 (CCK-8) assay following the manufacturer's specification (MedChem Express).

1.12. *In vitro* proliferation and osteogenic differentiation of rBMSCs

Mouse bone marrow mesenchymal stem cells (rBMSCs) have been extensively used to evaluate

cytocompatibility of biomaterials. Therefore, this cell line was also employed in the study. The rBMSCs were isolated from new-born Sprague Dawley (SD) rats (1 week) and cultured in Dulbecco's Modified Eagle Medium (DMEM) medium, which contains 100 U/mL penicillin, 0.1 mg/mL streptomycin and 10% FBS (Bosco Biotechnology Co., Ltd, China). The rBMSCs cells were co-cultured with scaffolds at a density of 2 × 10^4 cells per cell in 24-well culture plate. After 3 days of co-culture under NIR irradiation (0.45 W/cm^2, 41 ± 0.5 °C for 60 s), cells were stained by FDA/PI blend dyes and Rhodamine-labeled Phalloidin and DAPI dyes. The proliferation of cells was investigated by CCK-8 assay at 1, 3, 5, and 7 days, respectively.

After the co-culture of rBMSCs with scaffolds for 2 days, the culture medium was refreshed with the osteogenic medium (DMEM with 10% FBS, 1% penicillin/streptomycin, 50 μM/mL ascorbic acid, 0.1 μM dexamethasone, and 10 mM β-glycerophosphate) every 2 days. Meanwhile, photothermal groups were subjected to the periodical NIR irradiation every 3 days (0.45 W/cm^2, 41 ± 0.5 °C, and 60 s each time). After osteogenic culture for 7 and 14 days, the total RNA of rBMSCs was extracted by the Trizol reagent and reversely transcribed into cDNA. The primer sequences were listed in Table S1. The gene expressions of alkaline phosphatase (ALP), type I collagen (COL 1A1), bone morphogenetic protein 2 (BMP-2), and osteocalcin (OCN) were measured by Real-time reverse transcription-polymerase chain reaction (RT-PCR). In addition, the secretion of proteins mentioned above were also quantitatively detected following the protocols of enzyme-linked immunosorbent assay (ELISA).

1.13. Study on the mechanism of photothermal osteogenesis

1.13.1 Transcriptome sequencing

RNA-sequencing analysis was used to evaluate the expression of mRNA profiles in rBMSCs for the photothermal treated group (scaffold + NIR group, rBMSCs co-cultured with CS/PCL/BP/PDA@Ag scaffold under NIR irradiation) and the non-photothermal treated group (scaffold group, rBMSCs co-cultured with CS/PCL/BP/PDA@Ag scaffold). Briefly, rBMSCs were cultured in 24-well plates with CS/PCL/BP/PDA@Ag scaffolds for 7 days. photothermal

treated group was subjected to the periodical NIR irradiation every 3 days (0.45 W/cm^2, 60 s each time). On the 7th day, the collected cells were washed 3 times with PBS and then Rrizol (1 mL) was added to lysis the cells in a 1.5 mL EP tube. The enriched mRNA was fragmented and then reverse transcripted into cDNA with random primers. After cDNA fragments were ligated to illumina sequencing adapters, the ligation products were amplified and sequenced using illumina NovaSeq 6000（illumina, USA）by Novogene Biotech Co., Ltd. (Beijing, China).

DESeq2 software was used to determine the differentially expressed genes (DEGs) between photothermal treated group and non-photothermal treated group. Corrected P-value of 0.05 and absolute fold change (FC) of 2 were set as the threshold for significantly differential expression. Besides, Gene Ontology (GO) enrichment analysis and Kyoto Encyclopedia of Genes and Genomes (KEGG) pathway enrichment were implemented by the clusterProfiler R package to evaluate the molecular or biological functions of DEGs and identify enriched metabolic pathways in DEGs comparing with the whole genome background. Therein, Corrected P-value < 0.05 was considered significantly enriched by DEGs.

1.13.2 Heat shock proteins assays *in vitro*

The rBMSCs cultured on scaffolds were periodically subjected to NIR irradiation every 3 days (41 ± 0.5 ℃, and 60 s each time). After osteogenic culture for 5 hours and 7 days, the total RNA of rBMSCs was reversely transcribed into cDNA. The gene expressions of Hsp 47 and Hsp 70 (primers listed in Table S1) were quantitatively examined by RT-PCR.

1.14. Antibacterial assay *in vitro*.

Gram-negative *E. coli* (ATCC25922) and Gram-positive *S. aureus* (ATCC25923) were used to evaluate the antibacterial properties of the CS/PCL/BP/PDA@Ag scaffolds with different initial concentrations of Ag^+. The agar diffusion test was used to assess the release antibacterial properties of Ag-loaded scaffold. 100 μL bacterial suspensions (1×10^6 CFU/mL) were added

into Luria-Bertani (LB) agar medium, and then specimens were placed in an agar plate and cultured for 24 hours in incubator at 37 °C. The bacterial inhibition ratio was employed to quantitatively assess the antibacterial effects of the Ag-loaded scaffold. Typically, scaffold specimens were immersed in 1×10^5 CFU/mL bacterial suspensions for 24 hours. The medium without the bacterial solution and the bacterial solution without any treatment were set as blank and negative controls, respectively. Each test was performed with three parallel samples. At each specific time, bacterial suspensions were subjected to turbidity analysis by a digital camera and the optical density was also detected at 600 nm by using a microplate reader (Spectra Max ABS, USA). At the same time, 100 μL of each diluted fluid sample was inoculated on a nutrient Luria-Bertani agar plate. The number of colony-forning unit (CFU) was counted. The bacterial inhibition rate was calculated according to the equation.

$$\text{Bacterial inhibition rate (\%)} = (B_{Control} - B_{Test}) / B_{Control} \times 100\% \qquad (6)$$

Where $B_{Control}$ and B_{Test} were the OD values of the negative control group and the test groups, respectively.

The bacterial morphology on the scaffolds were also observed by SEM to evaluate the antibacterial effects of the scaffold. In detail, 1×10^6 CFU/mL bacterial suspensions were inoculated on the scaffold, and the scaffold was taken out and washed with PBS after 24 hours of culture. The scaffold was then fixed with glutaraldehyde, dehydrated with gradient ethanol and dried in air. Finally, the scaffold was subjected to gold sputtering for SEM observation.

1.15. Animal model of femoral defect infected with bacterial.

The Sprague Dawley (SD) rats (280 - 350 g, male) were commercially obtained from Chengdu Dossy Experimental Animals Co., Ltd (Chengdu, China), and fed in the animal facility with Specific Pathogen Free (SPF) conditions. All animal studies were performed with the permission of the Animal Research Committee of Sichuan University (approval number: 2019065A).

After acclimatizing for 7 days, general anesthesia was provided by intraperitoneal injection of

pentobarbital sodium (2%, 40 mg/kg). A cylindrical critical-defects at the size of Φ 2.5 mm × 3 mm were established to the hind leg femurs of SD rats with a 2.5 mm drill. Afterwards, all of them were injected with 1 × 10^6 CFU/mL of *S. aureus* bacterial suspension carefully. The hind legs of each rat were bilaterally implanted with the CS/PCL scaffold and CS/PCL/BP/PDA@Ag scaffold (2.5 mm diameter and 3 mm height). The contingent gaps were padded with bone wax and the wounds were closed carefully. The rats were randomly assigned to four groups to study the effects of infected defect repair as group 1: CS/PCL scaffold, group 2: CS/PCL scaffold + weekly NIR of post-surgery, group 3: CS/PCL/BP/PDA@Ag scaffold, and group 4: CS/PCL/BP/PDA@Ag scaffold + weekly NIR of post-surgery. After 8 and 12 weeks of surgical operation, the femurs were harvested and fixed in 4% polyformaldehyde for subsequent micro-computed tomography (micro-CT) imaging and histological assessment. Please refer to the details in *section 1.17. Microcomputed tomography (Micro-CT) test and histological assessment*.

1.16. *In vivo* anti-infection assay of the chloroplast-inspired scaffold.

In order to indicate degrees of anti-infection of different groups, the rats were assigned to another four groups: Blank (pure defects without injection of bacterium suspension), Control (pure defects with injection of bacterium suspension), infected bone defects with CS/PCL and CS/PCL/BP/PDA@Ag implantation. The infected tissues were harvested after 1, 4, 7 and 14 days of implantation, and corresponding inflammatory factors including rat interleukin 1β (IL-1β), interleukin 6 (IL-6) and tumor necrosis factor (TNF-a) were analyzed using an ELISA Kit (Bioswamp, China). Moreover, histological evaluation of inflammatory level immune response was measured by hematoxylin-eosin (H&E) staining. All the slices were observed through an optical microscope.

1.17. Microcomputed tomography (Micro-CT) test and histological assessment.

The animals were euthanized with an overdose of anesthetic at predetermined time points, and their femurs were harvested for Micro-CT and histological assessment. All femur specimens

were reconstructed with 3D and 2D geometries by VGS tudio Max software after 8 and 12 weeks of feeding. Cylinders with 2.5 mm diameter and 3 mm depth was selected to cover defect areas as the region of interest (ROI). Therefore, relevant images of frontal, transverse views and new bone growing tunnel into the defects were created within ROI region. Besides, bone mineral density (BMD), trabecular thickness (Tb, Th), trabecular number (Tb. N) and bone volume/tissue volume (BV/TV) were calculated for quantitative analysis of bone formation within the ROIs. Corresponding slices were stained with Masson trichrome staining and hematoxylin and eosin (H&E) staining. Furthermore, osteogenesis-related protein and their gene expressions were also tested by immunohistochemical staining and reverse transcription-polymerase chain reaction (RT-PCR) such as bone morphogenetic protein-2 (BMP-2), type I collagen (COL I) and osteocalcin (OCN). Primer sequences of the genes are provided in Table S2.

1.18. Statistical analysis.

Independent Student's t-test was executed to show the differences between groups, and all the numerical values were displayed as the mean ± standard deviation. $*p < 0.05$, $**p < 0.01$, and $***p < 0.001$ were considered as the statistic difference level. Each experiment included at least three replicates.

2. Supplementary Figures

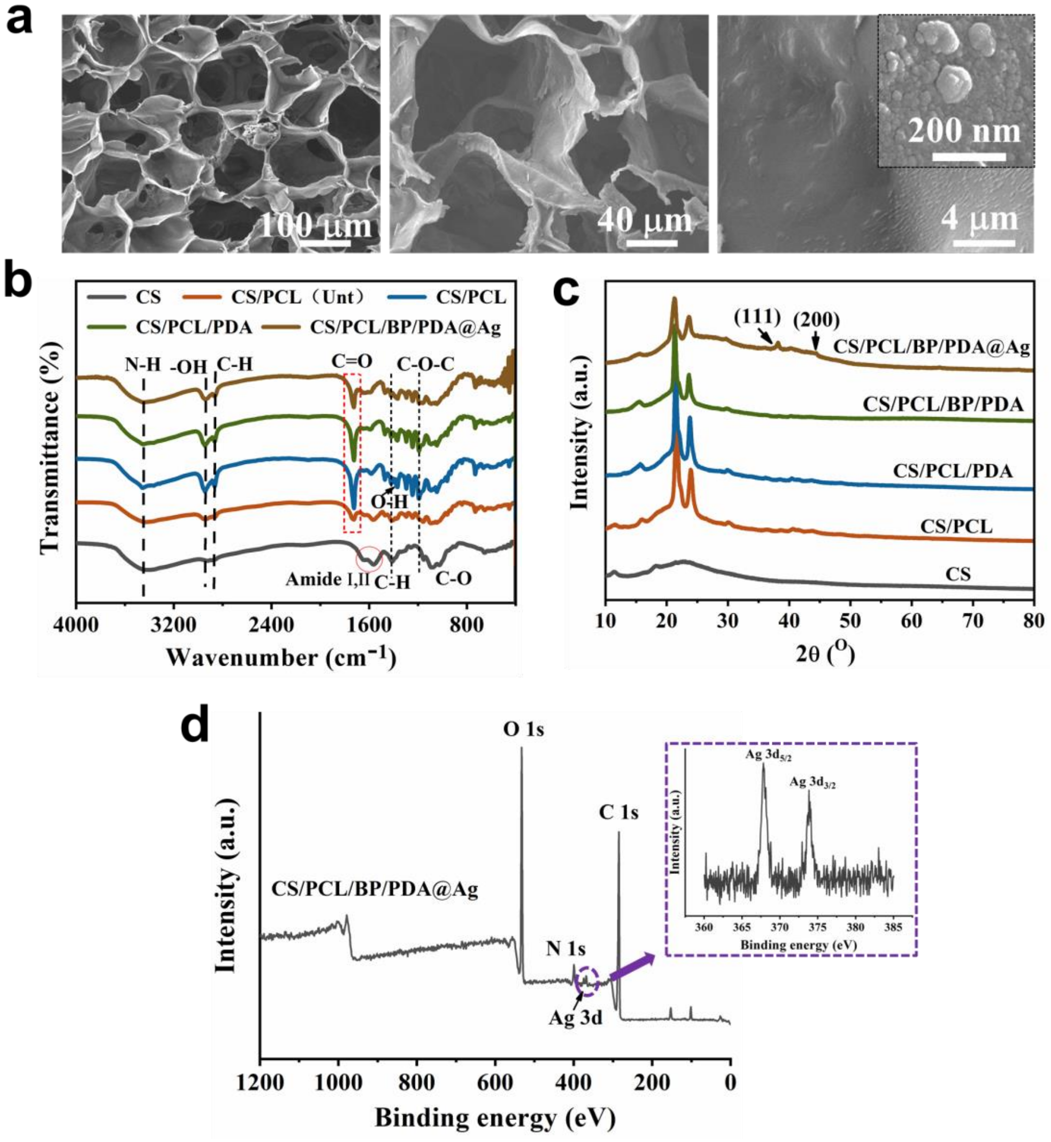

Figure S1. Characterization of scaffolds. a) The surface SEM images of the CS/PCL/BP/PDA@Ag scaffolds. Inset shows the image of Ag nanoparticles (scale bar = 200 nm). b) FTIR and c) XRD spectra of the corresponding scaffold. d) XPS spectra of the CS/PCL/BP/PDA@Ag scaffold. Inset in d) shows the high-resolution spectra of the Ag 3d in the CS/PCL/BP/PDA@Ag scaffold.

Chemical structure of the scaffolds was analyzed by FTIR spectra (Figure S1b). The stretching vibrations of the methylene ($-CH_2$, 2867 cm^{-1}), the stretching vibrations of the protonated amine group (3457 cm^{-1}) and the characteristic peaks of amide I and amide II (1646 cm^{-1} and 1560 cm^{-1}) mainly come from CS.[3] The characteristic peak of 3433 cm^{-1} in all group was the stretching vibrations of the hydroxyl (-OH) stemmed from CS and PCL. It's worth noting that

the typical peak of carbonyl (C=O, 1712 cm^{-1}) appears in CS/PCL, CS/PCL/PDA and CS/PCL/BP/PDA@Ag scaffold (heighted by the red rectangles), whereas it did not appear in CS/PCL (Unt) scaffold, indicating that PCL had been successfully modified with carboxyl in CS/PCL, CS/PCL/PDA and CS/PCL/BP/PDA@Ag scaffold.

Crystal structure analysis with XRD found that the diffraction peak at 22.69° was derived from CS powder, which showed a typical amorphous structure. The diffraction characteristic peaks of PCL mainly appeared at 16°, 21.4° and 23.7°, and all of these peaks were presented in the CS/PCL, CS/PCL/PDA, CS/PCL/BP/PDA and CS/PCL/BP/PDA@Ag scaffold.[4] Furthermore, the diffraction characteristic peaks in CS/PCL/BP/PDA@Ag scaffold at 38.13° and 44.35° were corresponding to the (111) and (200) diffraction lattice planes of Ag nanoparticles,[5] respectively (Figure S1c). As a result, these results indicate that the Ag nanoparticles have been prepared *in situ* in the CS/PCL/BP/PDA@Ag scaffold.

The X-ray photoelectron spectroscopy (XPS) survey spectra of CS/PCL/BP/PDA@Ag scaffold was shown in Figure S1d. The characteristic peaks of carbon (C), oxygen (O), nitrogen (N) and silver (Ag) elements were detected in the scaffold, proving the existence of Ag nanoparticles. The high-resolution spectra showed that the binding energy of Ag 3d mainly included two peaks of Ag $3d_{5/2}$ and Ag $3d_{3/2}$, which are located at 368.83 eV and 374.52 eV, respectively (heighted by the purple rectangles).[6] All of the results forcefully confirmed that Ag nanoparticles have been successful mineralized *in situ* into the scaffold, which is consistent with the results of XRD in Figure S1c.

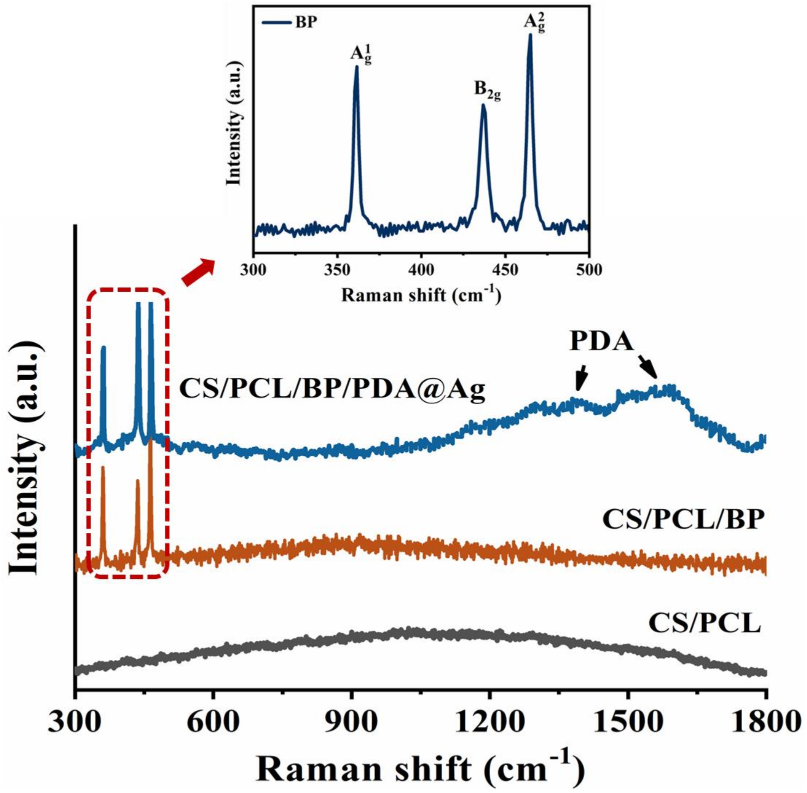

Figure S2. Raman spectra acquired from the CS/PCL, CS/PCL/BP and CS/PCL/BP/PDA@Ag scaffold, respectively. Inset shows the BP spectra.

XPS spectra above only showed the characteristic peak of Ag, whereas that of BP had not been found. In general, XPS is mainly used to detect the composition of elements on the surface of the bulk materials with a few nanometers, while the absence of the characteristic peaks of BP maybe because of the coating effect of polydopamine layer. Therefore, Raman measurement was employed to clarify the existence of BP because of the intense signal of conjugate structures in BP. As shown in Figure S2, compared with the CS/PCL scaffold, the Raman spectrums of the CS/PCL/BP and CS/PCL/BP/PDA@Ag scaffold appear three sharp peaks. The three sharp peaks were attributed to the out-of-plane phonon mode ($A^1{}_g$) at 359.5 cm^{-1} as well as two in-plane modes (B_{2g} and $A^2{}_g$) at 436.0 and 463.3 cm^{-1}, respectively.[7] In addition, the two broad peaks at 1385.7 cm^{-1} and 1574.9 cm^{-1} mainly originated from the stretching and deformation of aromatic rings of PDA in CS/PCL/BP/PDA@Ag scaffold. Therefore, the existence of BP in as-designed scaffold has been successfully proved.

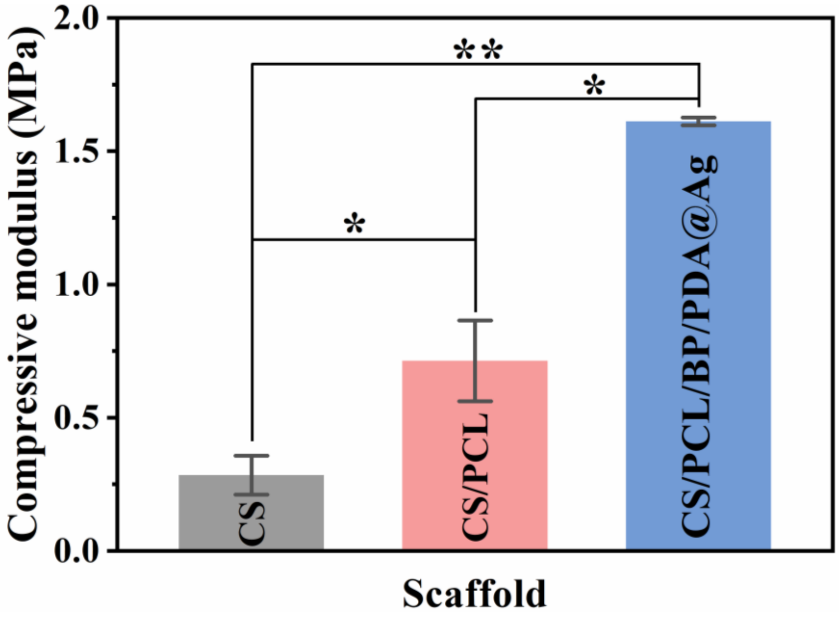

Figure S3. Analysis of elastic modulus of CS, CS/PCL and CS/PCL/BP/PDA@Ag scaffold. Error bars: mean ± SD (n = 5), *p < 0.05, **p< 0.01.

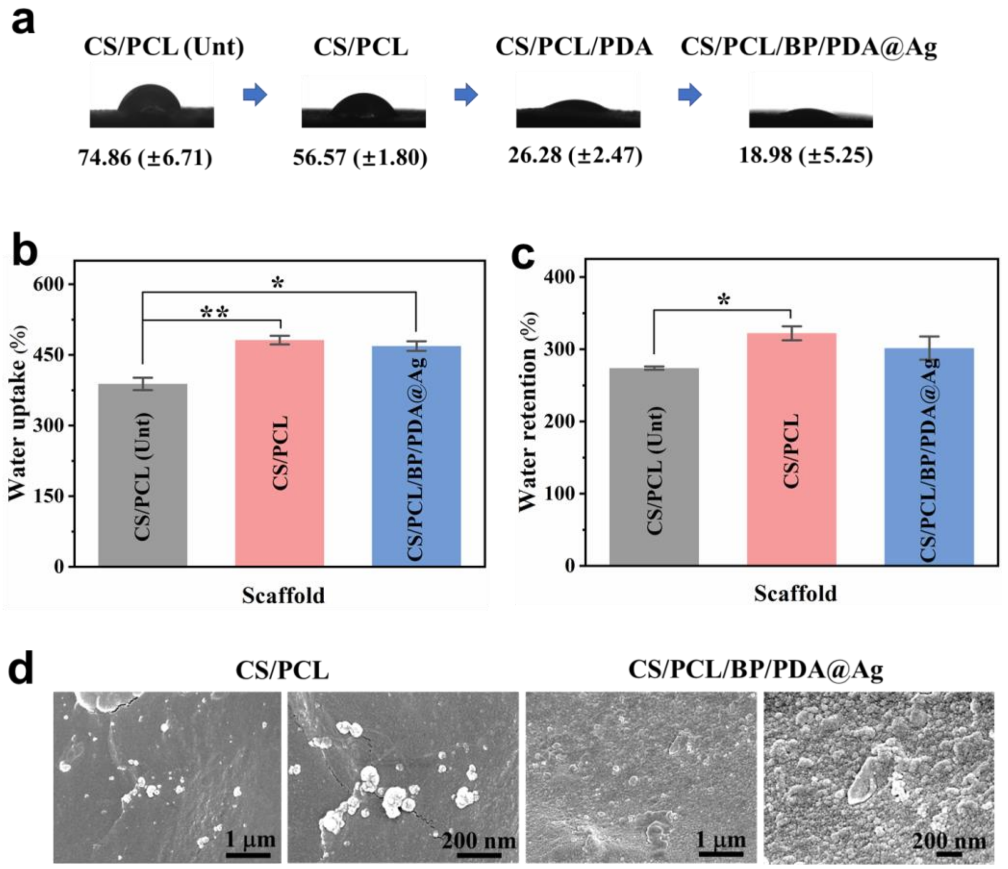

Figure S4. Study on the hydrophilicity of scaffolds. a) The variation of water contact angle on different sample surfaces. b) Water uptake and c) retention ratio of the scaffolds immersed in distilled water for 48 hours. d) SEM images of the scaffolds after biomineralization in simulated body fluid (SBF) for 1 day. Error bars: mean ± SD (n = 5), *$p < 0.05$, **$p < 0.01$.

The hydrophilicity of untreated CS/PCL scaffold (CS/PCL (Unt)) could be improved by functionalizing with carboxyl (Figure S4a). After the surface modification with PDA coating, the hydrophilicity of CS/PCL/PDA and CS/PCL/BP/PDA@Ag scaffolds could be further improved. Based on this, the CS/PCL/BP/PDA@Ag scaffold would absorb much water to swell. Nevertheless, there was no obvious difference between CS/PCL and CS/PCL/BP/PDA@Ag scaffold in swelling during the entire test period. The water uptake (Figure S4b) and water retention ratio (Figure S4c) both in CS/PCL and CS/PCL/BP/PDA@Ag scaffolds were higher than these in CS/PCL (Unt) scaffold. Because the surface of CS/PCL scaffold was covered with abundant carboxyl functional groups, the hydrophilicity of PCL was improved, and further its water uptake ratio and water retention ratio were enhanced as well. However, there was no

significant difference between CS/PCL/BP/PDA@Ag and CS/PCL scaffold, indicating that PDA stabilized BP and Ag nanoparticles in CS/PCL/BP/PDA@Ag scaffold had little effect on the water retention ratio compared to CS/PCL scaffold. After just one day of immersion in SBF, some granular nanoparticles grow on the surface of both CS/PCL scaffold and CS/PCL/BP/PDA@Ag scaffold (Figure S4d). However, more nanoparticles were formed on the CS/PCL/BP/PDA@Ag scaffold than on the CS/PCL scaffold.

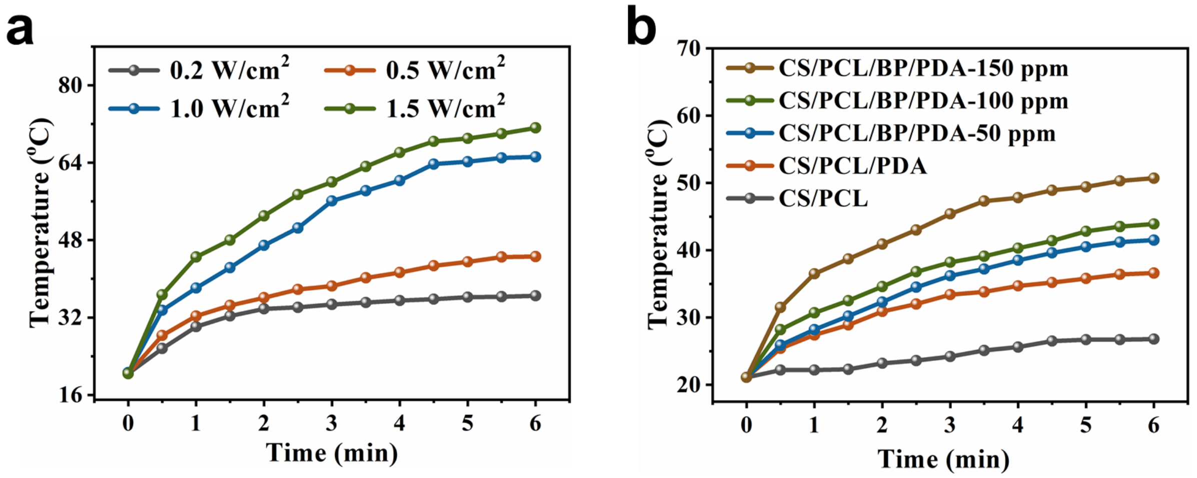

Figure S5. Photothermal properties of the scaffolds. a) Temperature variation of CS/PCL/BP/PDA-100 ppm scaffold in PBS under laser irradiation with different laser power densities (0.2, 0.5, 1.0 and 1.5 W/cm^2). b) Photothermal heating curves of the CS/PCL, CS/PCL/PDA and CS/PCL/BP/PDA scaffolds with various BP concentrations irradiated by a NIR laser (808 nm, 0.5 W/cm^2).

The photothermal properties of CS/PCL/BP/PDA scaffold were evaluated firstly via the immersion of all scaffolds in PBS. The temperature of the wet CS/PCL/BP/PDA scaffold rised quickly with the increase of laser power density (Figure S5a). The temperature of the CS/PCL scaffold showed a little increase after 6 min of irradiation (increase by 5.7 °C), which might result from the weak interaction of NIR laser and CS/PCL macromolecules. Besides, the temperature of CS/PCL/PDA scaffolds increased by 15.5 °C after 6 min of irradiation, which mainly stems from the inherent photothermal responsiveness of PDA. [8]

And the photothermal performance of the CS/PCL/BP/PDA scaffolds with different BP concentrations (50, 100 and 150 ppm) was also concentration-dependent owing to the intrinsic photothermal conversion efficiency of BP (Figure S5b).

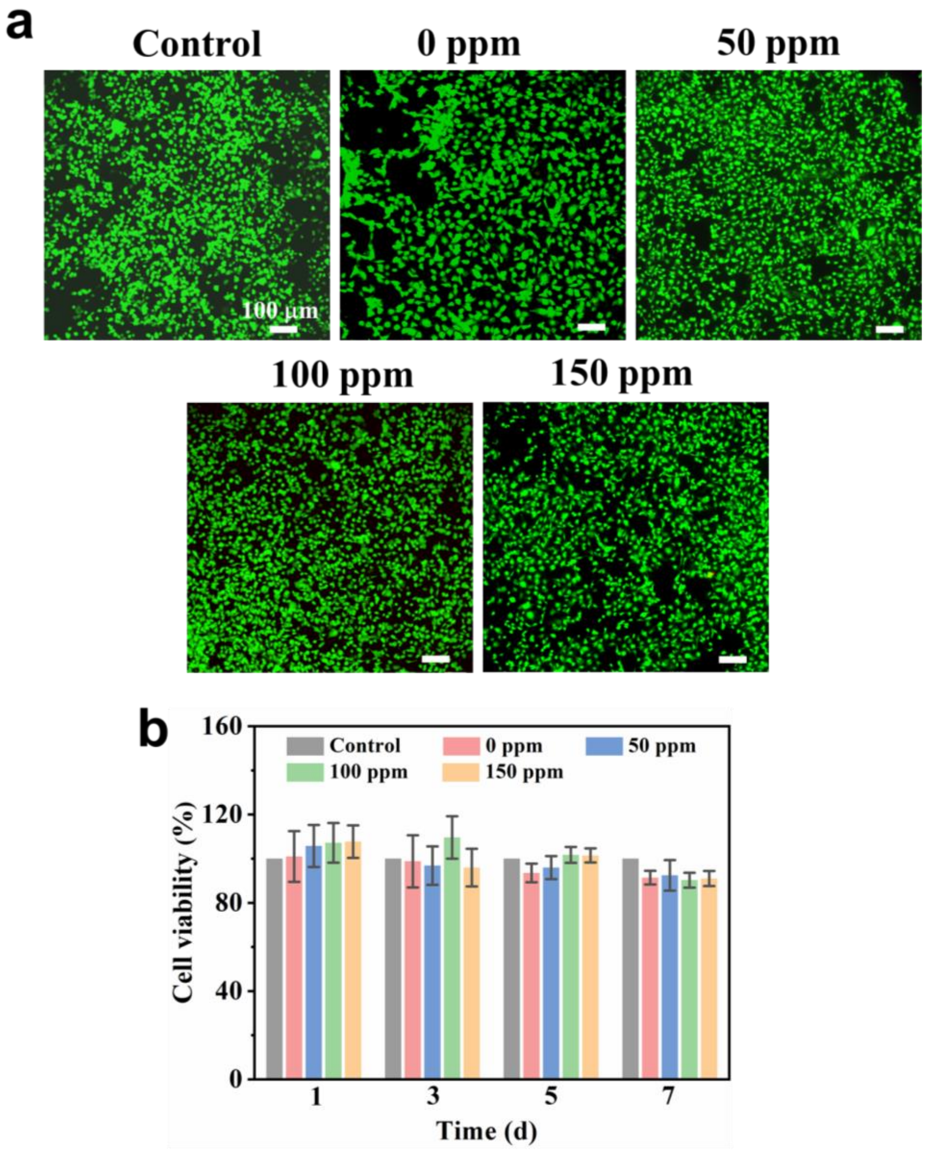

Figure S6. Assessment of cytocompatibility *in vitro*. a) Representative live/dead images of MC3T3-E1 cells incubated with control (without scaffold) and CS/PCL/BP/PDA scaffold with various concentrations of BP for 3 days, green represents living cells and red represents dead cells. b) The relative cell viabilities of MC3T3-E1 cells incubated with CS/PCL/BP/PDA scaffold with various concentrations of BP for 1, 3, 5, and 7 day (s).

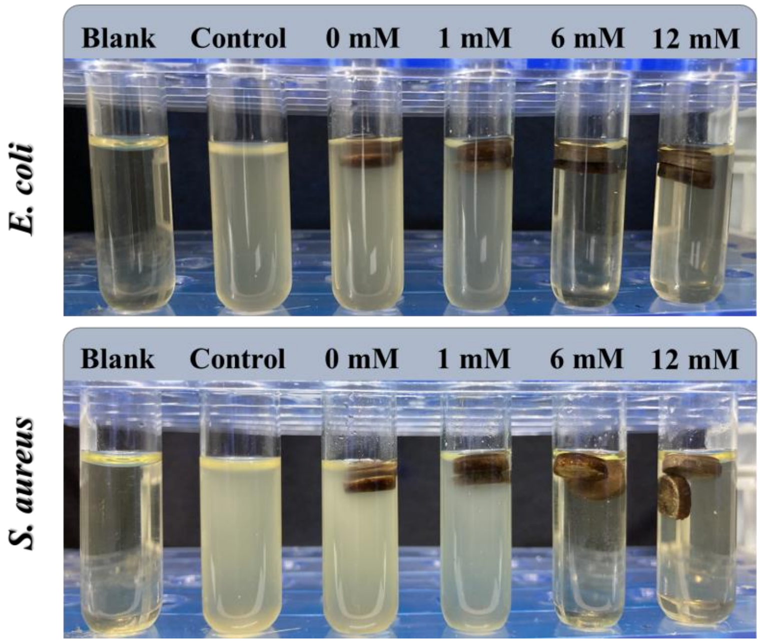

Figure S7. Turbidity analysis of bacterial suspensions co-cultured with the different CS/PCL/BP/PDA@Ag scaffolds after 24 hours.

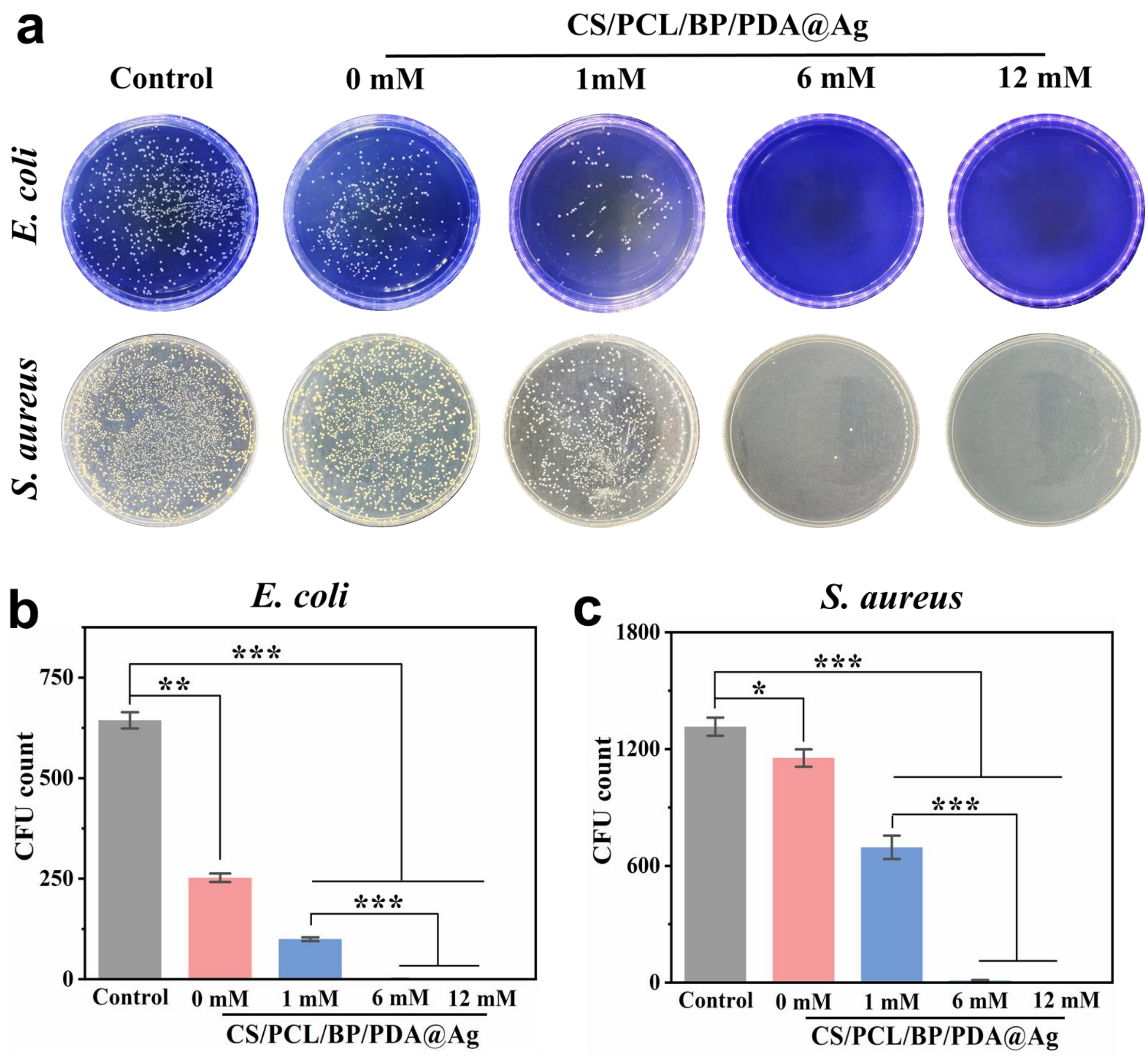

Figure S8. a) Agar plates of bacterial suspensions after co-cultured with different scaffolds for 24 hours, and the colony-forming unit (CFU) amount of b) *E. coli* and c) *S. aureus* in agar plates. Error bars: mean ± SD (n = 5), *$p < 0.05$, **$p < 0.01$, ***$p < 0.001$.

To further intuitively reflect the antibacterial effect, the CFU of diluted suspension obtained in agar plate was measured. As shown in Figure S8a, there was no or less colony observed on the solid agar plates in CS/PCL/BP/PDA@Ag-6 mM and CS/PCL/BP/PDA@Ag-12 mM groups, and the corresponding CFU quantitative count also illustrates the superior antibacterial property of CS/PCL/BP/PDA@Ag-6 mM and CS/PCL/BP/PDA@Ag-12 mM groups compared with CS/PCL/BP/PDA@Ag-1 mM and Ag-free groups (Figure S8b and c).

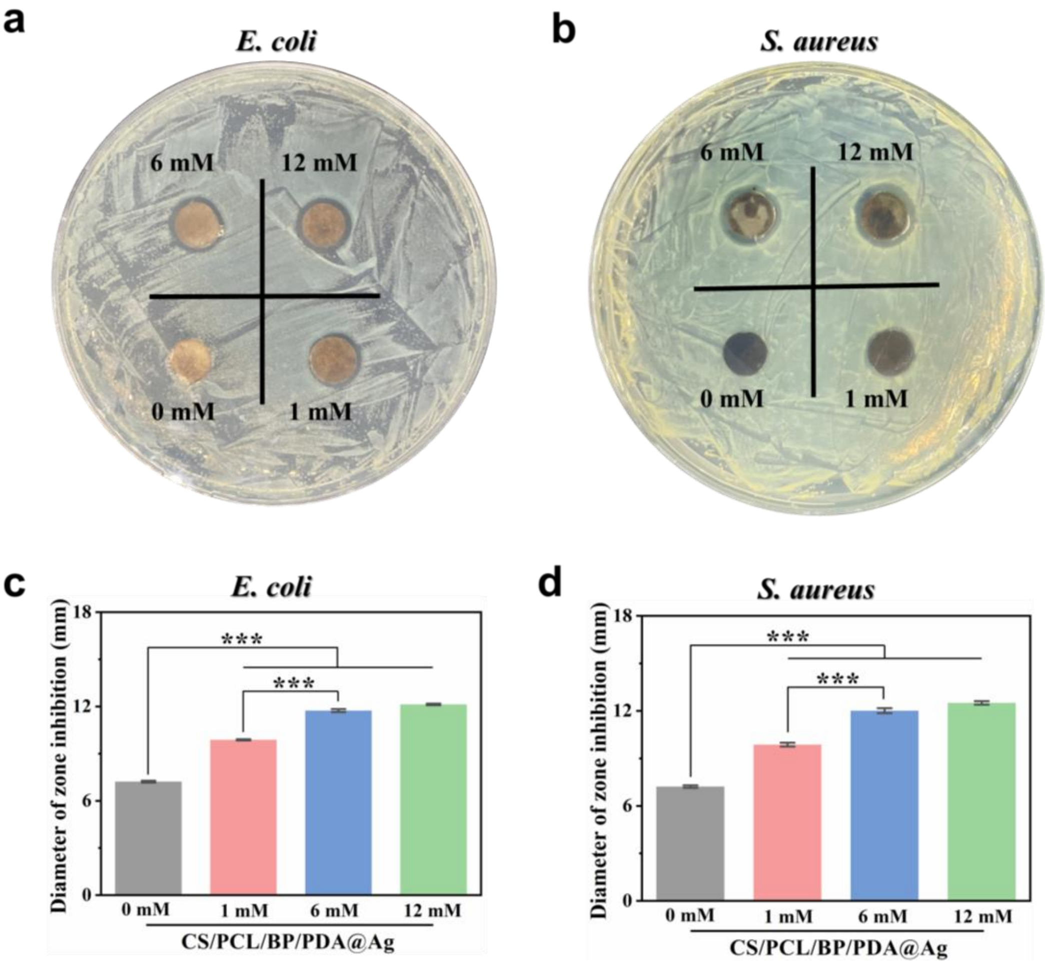

Figure S9. Analysis of the size of inhibition zone of different scaffolds against *E. coli* and *S. aureus* in agar plates. Macroscopic inhibition rings formed around the CS/PCL/BP/PDA@Ag scaffolds against a) *E. coli* and b) *S. aureus* in agar plates after 24 hours of culture. The corresponding cartograms show the qualitative size of inhibition zones of different scaffolds against c) *E. coli* and d) *S. aureus*. Error bars: mean ± SD (n = 5), ****p* < 0.001.

The CS/PCL/BP/PDA@Ag scaffolds without decorating of Ag nanoparticles did not exhibit any inhibition zone, indicating no antibacterial ability towards *E. coil* and *S. aureus* (Figure S9a and b). In contrast, the CS/PCL/BP/PDA@Ag scaffold with different initial Ag^+ concentrations showed a visible inhibition zone, illustrating that the surrounding bacteria were killed owing to the diffusion of Ag^+ from the CS/PCL/BP/PDA@Ag scaffolds. The diameter of the inhibition zones for *E. coil* and *S. aureus* (Figure S9c and d) was also quantified. It was obviously found that the maximal inhibition zone appeared in the CS/PCL/BP/PDA@Ag-12 mM group for both *S. aureus* (12.5 ± 0.02 mm) and *E. coil* (12.12 ± 0.05 mm), indicating the robust antibacterial property.

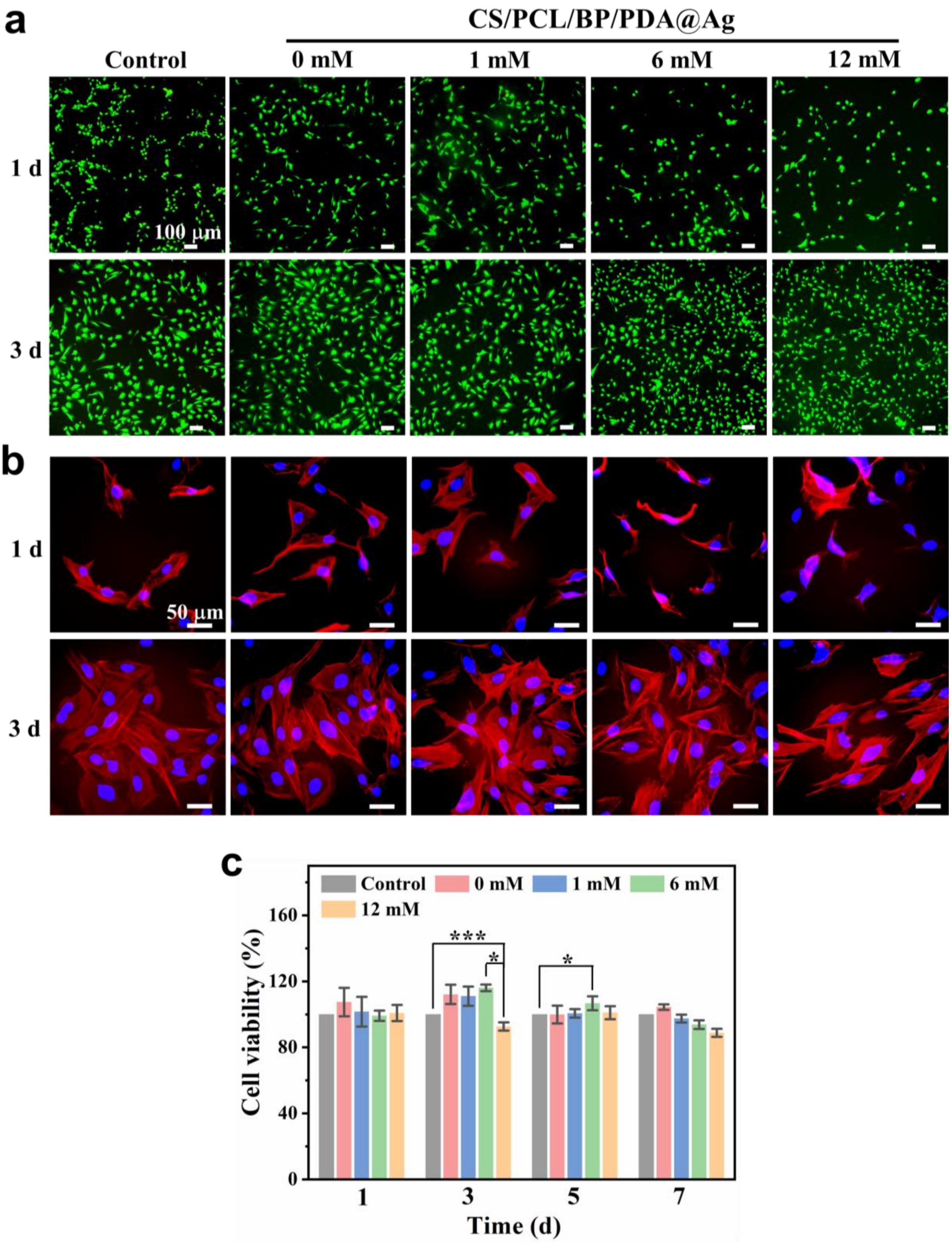

Figure S10. Assessment of cytocompatibility *in vitro*. a) Representative live/dead images of MC3T3-E1 cells incubated with control (without scaffold) and chloroplast-inspired CS/PCL/BP/PDA@Ag scaffolds, green represents living cells and red represents dead cells. b) Fluorescence staining of cellular morphology, F-actin is stained in red and nucleus is stained in blue. c) CCK-8 assay for the cell proliferation cultured with different extracts of scaffold for 1, 3, 5, and 7 days. Error bars: mean ± SD (n = 5), *p< 0.05, ***p< 0.001.

For *in vivo* implant biomaterials, cytocompatibility is a key step that must be considered. Hence, cytocompatibility of scaffolds *in vitro* was assessed (Figure S10). The viability of MC3T3-E1 cells on CS/PCL/BP/PDA@Ag scaffolds was studied by Live & Dead staining at 1 and 3 days (Figure S10a). Fluorescent staining images of live/dead cells showed that the living cells dominated in all groups, and the amount of living cells increased gradually from 1 day to 3 days. Only a small number of dead cells were found in CS/PCL/BP/PDA@Ag-12 mM group at 3 days. In order to investigate the cellular morphology, the actin and nucleus of cells was stained in red and blue by rhodamine-phalloidin and DAPI assay, respectively (Figure S10b). The osteoblasts exhibited spindle-shaped morphology in all CS/PCL/BP/PDA@Ag groups, which are consistent with the characteristic shapes of MC3T3-E1 cells.[3] Cellular quantity kept an increasing trend during incubation with scaffolds. Besides, cell proliferation was also estimated by CCK-8 assay for 1, 3, 5, and 7 days (Figure S10c). The viability of osteoblasts in all groups was more than 85%, which satisfied the requirement of cytocompatibility for biomaterials.

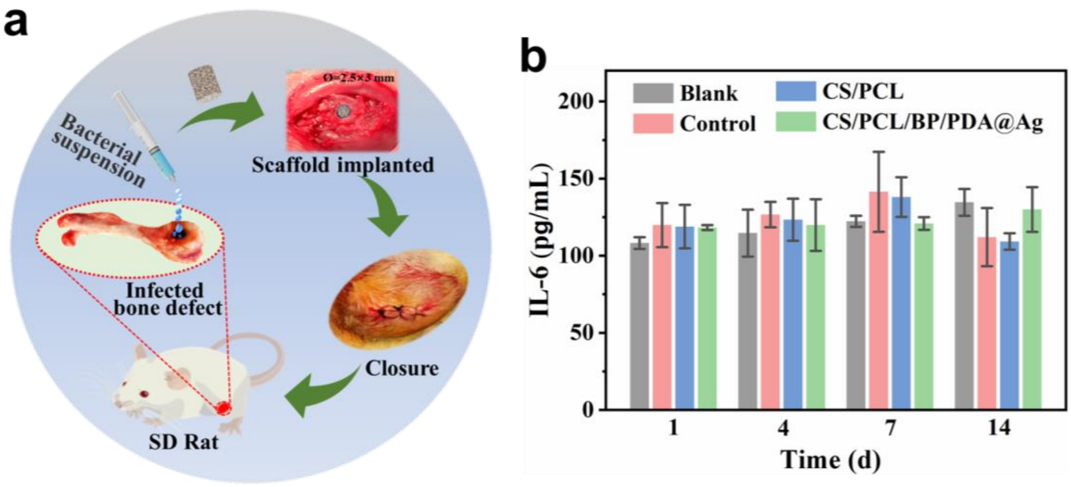

Figure S11. a) Schematic diagram showing the progress of implementation of SD rat femoral defect model infected with *S. aureus* and related scaffolds implantation. b) Concentration variation of related inflammatory factors of rat interleukin 6 (IL-6) during postoperative infection (Blank represents uninfected bone defect without any treatment. Control represents infected bone defect without any treatment). Error bars: mean ± SD (n = 5).

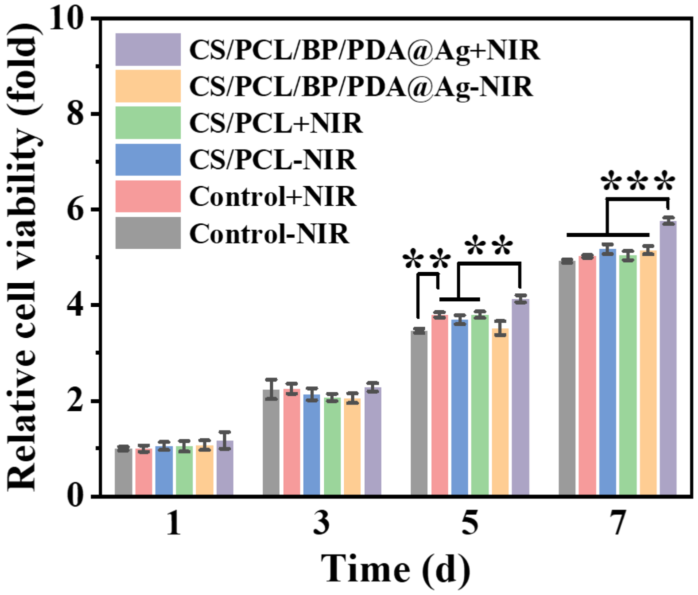

Figure S12. The CCK-8 assay for the cell proliferation co-cultured with different scaffolds under NIR irradiation or non-irradiation for 1, 3, 5, and 7 days. Error bars: mean ± SD (n = 5), ***p*< 0.01, ****p*< 0.001.

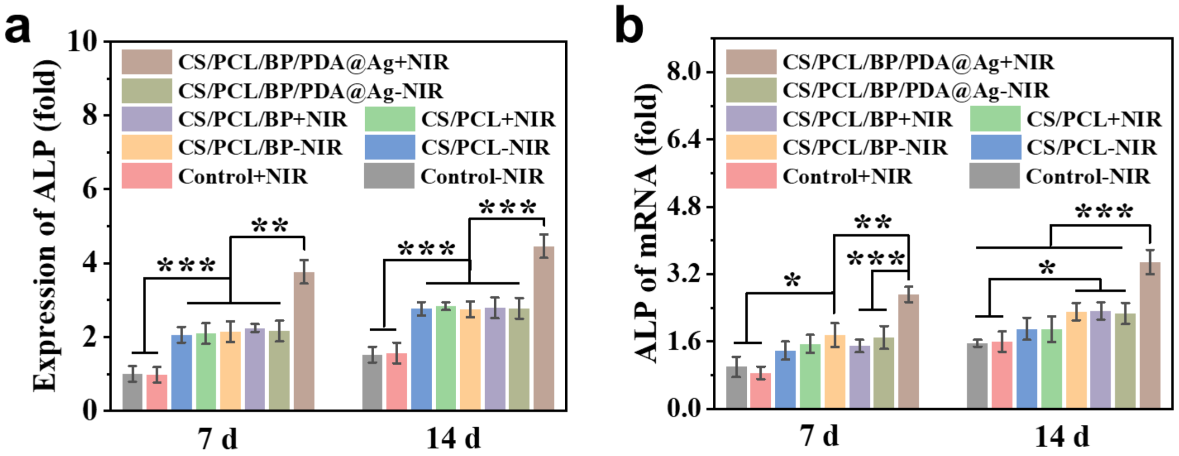

Figure S13. The osteogenic differentiation of rBMSCs on CS/PCL, CS/PCL/BP and CS/PCL/BP/PDA@Ag scaffolds with or without periodical NIR irradiation. The expressions of ALP a) proteins and b) genes in rBMSCs after osteo-induction for 7 and 14 days (taking control - NIR group as reference). Error bars: mean ± SD (n = 5), **p*< 0.05, ***p*< 0.01, ****p*< 0.001.

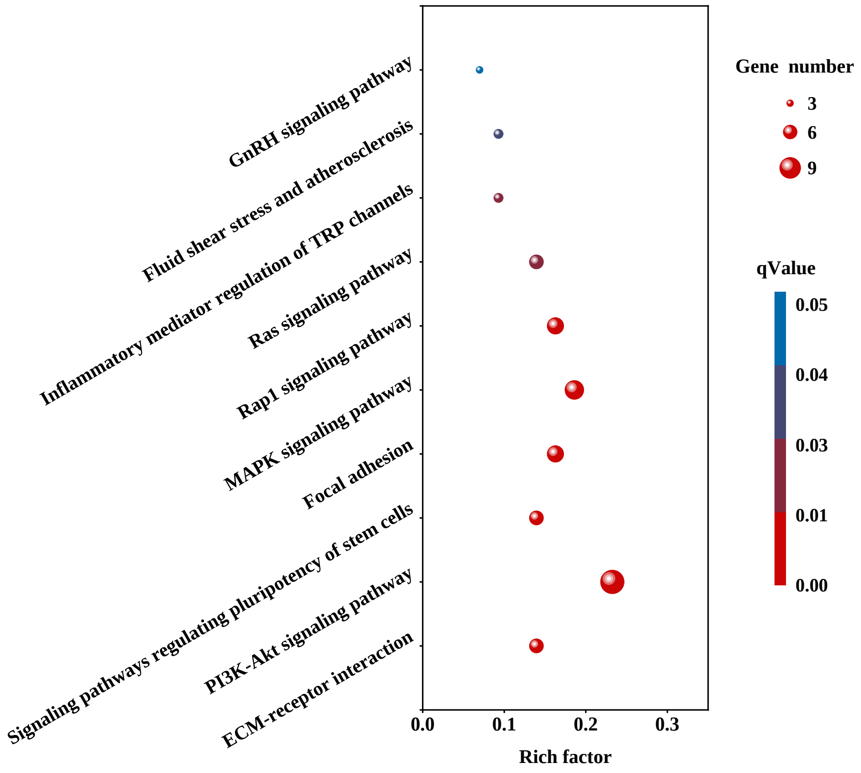

Figure S14. KEGG analysis revealing pathways and processes enriched in photothermal treated group (scaffold + NIR).

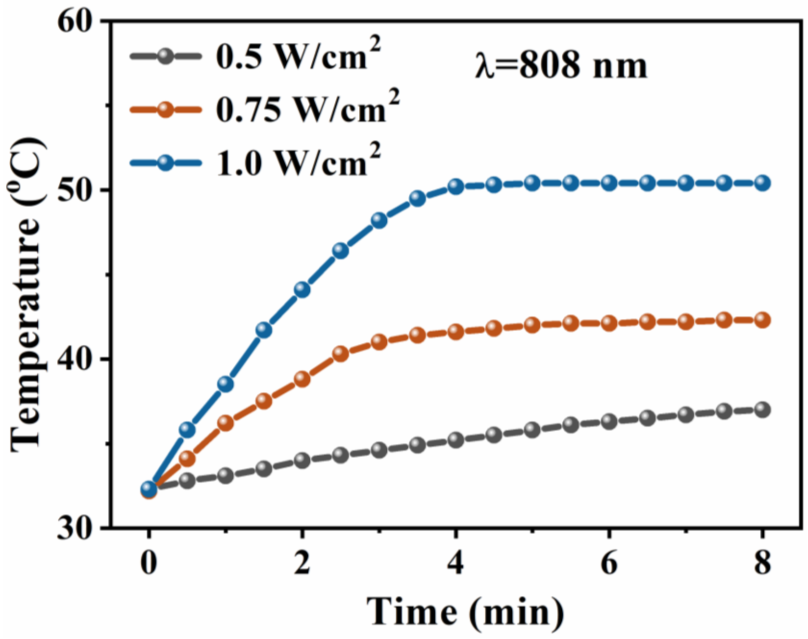

Figure S15. Temperature variation of CS/PCL/BP/PDA@Ag scaffold in the rat femoral defect *in vivo* under laser irradiation with different laser power densities (λ=808 nm, 0.5, 0.75 and 1.0 W/cm^2).

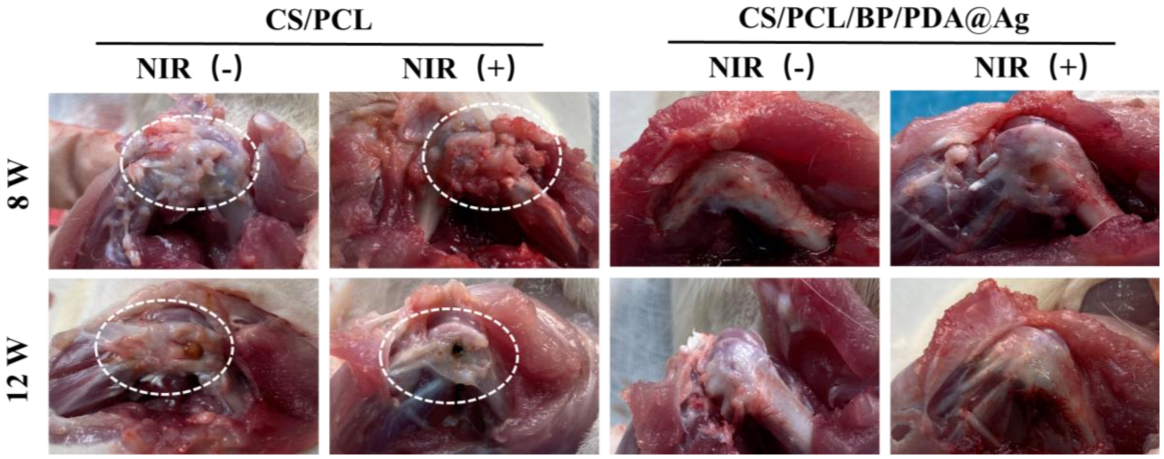

Figure S16. Representative macroscopic images of the femur specimens. White dashed circles highlights the festering areas.

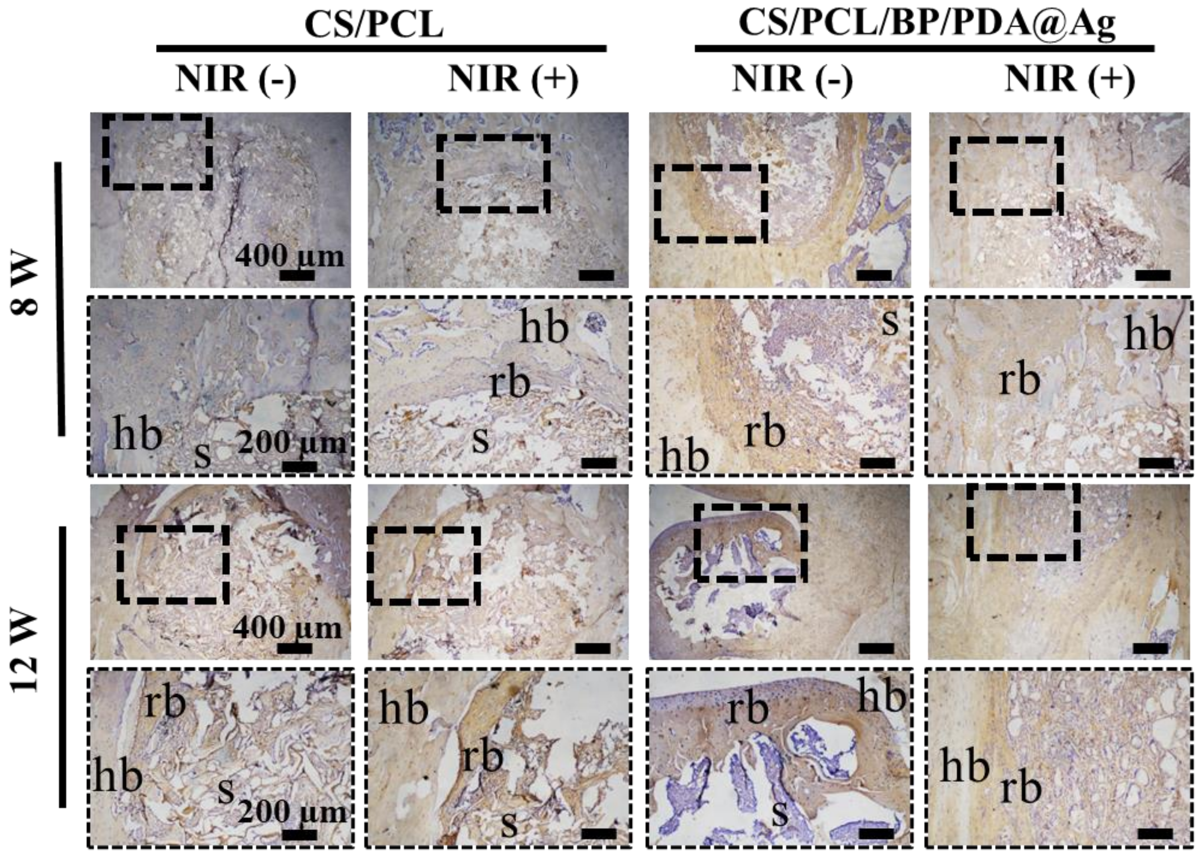

Figure S17. Immunohistochemical staining of bone-specific proteins after 8 and 12 weeks of implantation with or without periodical NIR irradiation. Positive brown staining of BMP-2 productions in rat femoral defects implantation under different magnification photomicrographs. Meanwhile, hb, host bone. rb, regenerated bone. s, scaffold. The enlarged areas are marked with rectangular dashed line.

Table S1. Primer sequences utilized for real time RT-PCR *in vitro* study.

Target gene	Forward primer sequence	Reverse primer sequence
ACTB	GAAGATCAAGATCATTGCTCCT	TACTCCTGCTTGCTGATCCACA
ALP	CATCATGTTCCTGGGAGATG	GGTGTTTGTACGTCTTGGAGA
OCN	GTAAGGTGGTGAATAGACT	GGGTTGAGCTCACACACCT
COL1A1	TGGCGCTTCAGGTCCAAT	TGTTCCAGGCAATCCACGAG
BMP-2	CGGTCTCCTAAAGGTCGACCAT	CGAACTTCTTGCGGCCCAGCT
HSP70	CCCAGATCGAGGTGACCTT	ACCATGCGCTCGATCACCT
HSP47	CTCCAGCCTCATCATCCTCAT	CTGCAGGTCATGGGTCACCT

Table S2. Primer sequences utilized for real time RT-PCR *in vivo* study.

Target gene	Forward primer sequence	Reverse primer sequence	Probe primer sequence
ACTB	GAAGATCAAGA TCATTGCTCCT	TACTCCTGCTTGCTG ATCCACA	TCACTGTCCACCTT CCAGCAGA
BMP-2	CGGTCTCCTAAA GGTCGACCAT	CGAACTTCTTGCGG CCCAGCT	CTGCTTCCCCAGGT CCTCCTG
CoL1A1	GTAAGGTGGTG AATAGACT	GGGTTGAGCTCACA CACCT	CCCAGATCCCCTGG AGCC
OCN	TGGCGCTTCAGG TCCAAT	TGTTCCAGGCAATC CACGAG	CCAGCTTCCCCATC ATCTCC

References

[1] X. Wang, J. Shao, M. Abd El Raouf, H. Xie, H. Huang, H. Wang, P. K. Chu, X.-F. Yu, Y. Yang, A. M. AbdEl-Aal, N. H. M. Mekkawy, R. J. Miron, Y. Zhang, *Biomaterials* **2018**, *179*, 164.

[2] Y. Zhao, J. Chen, L. Zou, G. Xu, Y. Geng, *Chem. Eng. J.* **2019**, *378*, 1.

[3] Y. Zhao, T. Fan, J. Chen, J. Su, X. Zhi, P. Pan, L. Zou, Q. Zhang, *Colloids Surf. B* **2019**, *174*, 70.

[4] S. Y. Tong, Z. Wang, P. N. Lim, W. Wang, E. S. Thian, *Mater. Sci. Eng. C* **2017**, *70*, 1149.

[5] C. Tong, X. Zhong, Y. Yang, X. Liu, G. Zhong, C. Xiao, B. Liu, W. Wang, X. Yang, *Biomaterials* **2020**, *243*, 1.

[6] X. Xie, T. Sun, J. Xue, Z. Miao, X. Yan, W. Fang, Q. Li, R. Tang, Y. Lu, L. Tang, Z. Zha, T. He, *Adv. Funct. Mater.* **2020**, *30*, 1.

[7] H. Fu, Z. Li, H. Xie, Z. Sun, B. Wang, H. Huang, G. Han, H. Wang, P. Chu, X. Yu, *RSC Adv.* **2017**, *7*, 14618.

[8] X. Zeng, M. Luo, G. Liu, X. Wang, W. Tao, Y. Lin, X. Ji, L. Nie, L. Mei, *Adv. Sci.* **2018**, *5*, 1.
